# A New Quinazolinone Alkaloid along with Known Compounds with Seed-Germination-Promoting Activity from *Rhodiola tibetica* Endophytic Fungus *Penicillium* sp. HJT-A-6

**DOI:** 10.3390/molecules29092112

**Published:** 2024-05-02

**Authors:** Dongliang Xiao, Yan Wang, Congcong Gao, Xuemei Zhang, Weixing Feng, Xuan Lu, Baomin Feng

**Affiliations:** College of Life and Health, Dalian University, Dalian 116622, China; xdl120318@163.com (D.X.); wangyan_9910@163.com (Y.W.); gcc1125@163.com (C.G.); zxuemei2024@163.com (X.Z.); fwx_0910@163.com (W.F.)

**Keywords:** *Rhodiola tibetica*, *Penicillium* sp. HJT-A-6, quinazolinone, seed-germination-promoting activity

## Abstract

A new quinazolinone alkaloid named peniquinazolinone A (**1**), as well as eleven known compounds, 2-(2-hydroxy-3-phenylpropionamido)-*N*-methylbenzamide (**2**), viridicatin (**3**), viridicatol (**4**), (±)-cyclopeptin (**5a**/**5b**), dehydrocyclopeptin (**6**), cyclopenin (**7**), cyclopenol (**8**), methyl-indole-3-carboxylate (**9**), 2,5-dihydroxyphenyl acetate (**10**), methyl *m*-hydroxyphenylacetate (**11**), and conidiogenone B (**12**), were isolated from the endophytic *Penicillium* sp. HJT-A-6. The chemical structures of all the compounds were elucidated by comprehensive spectroscopic analysis, including 1D and 2D NMR and HRESIMS. The absolute configuration at C-13 of peniquinazolinone A (**1**) was established by applying the modified Mosher’s method. Compounds **2**, **3**, and **7** exhibited an optimal promoting effect on the seed germination of *Rhodiola tibetica* at a concentration of 0.01 mg/mL, while the optimal concentration for compounds **4** and **9** to promote *Rhodiola tibetica* seed germination was 0.001 mg/mL. Compound **12** showed optimal seed-germination-promoting activity at a concentration of 0.1 mg/mL. Compared with the positive drug 6-benzyladenine (6-BA), compounds **2**, **3**, **4**, **7**, **9**, and **12** could extend the seed germination period of *Rhodiola tibetica* up to the 11th day.

## 1. Introduction

Significant mutualistic relationships have been established between endophytic fungi and their host plants, attracting considerable attention due to their ecological and biotechnological potential [1]. Endophytic fungi can produce a variety of secondary metabolites on their own and can be involved in the biosynthesis and biotransformation of secondary metabolites in host plants, making them an important source of active natural products. Active natural products derived from endophytic fungi hold vast potential applications in biopharmaceuticals, agricultural production, and industrial fermentation [2,3,4,5]. Currently, the secondary metabolites isolated from endophytic fungi fermentation products include alkaloids, polyketides, terpenes, etc. [2,6], and some even possess activity in promoting seed germination [7].

Quinazolinones have great development prospects in medicinal chemistry [8], originating from a wide range of antibacterial [9], anti-inflammatory [10,11], antiviral [12,13], and antituberculosis [14] properties. So far, only a limited number of quinazolinones have been reported from endophytic fungi, including neosartoryadins and glyantrypines, antiviral agents from the mangrove-derived fungi *Neosartorya udagawae* and *Cladosporium* sp., respectively [15,16]; chaetominine, a cytotoxic agent from the endophytic fungus *Chaetomium* sp. [17]; aniquinazolines A–D, antibacterial and cytotoxic molecules from the mangrove-derived fungus *Aspergillus nidulans* [18]; and (–)-(1*R*,4*R*)-1,4-(2,3)-Indolmethane-1-methyl-2,4-dihydro-1*H*-pyrazino-[2,1-*b*]-quinazoline-3,6-dione, an antifungal agent from the endophytic fungus *Penicillium vinaceum* [19]. This denotes that endophytic fungi still represent an underexploited reservoir of novel bioactive quinazoline molecules.

**Figure 1:**
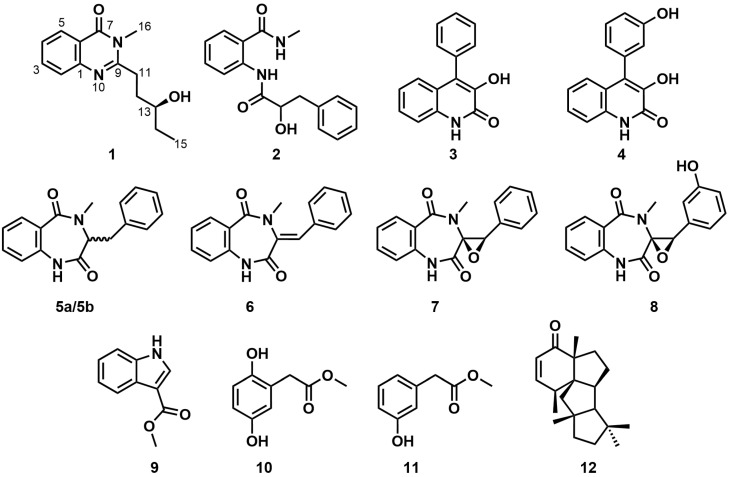
Chemical structures of compounds **1**–**12**.

Herein, as part of our ongoing studies on the bioactive secondary metabolites from *Rhodiola tibetica* endophytic fungi [20,21,22], we performed a Global Natural Products Social (GNPS) molecular networking analysis of the EtOAc extract of the endophytic fungus *Penicillium* sp. HJT-A-6. GNPS molecular networking has been widely applied in the analysis of natural products to cluster compounds with similar MS/MS spectra, expediting the dereplication process of known natural products [23,24,25]. The GNPS molecular networking analysis of the EtOAc extract led to the discovery of a new quinazolinone alkaloid, peniquinazolinone A (**1**), together with eleven known secondary metabolites (Figure 1). The seed-germination-promoting activities of the isolated compounds were also evaluated.

## 2. Results and Discussion

### 2.1. Molecular Networking-Guided Isolation Workflow

To target the isolation of the quinazolinone alkaloid, the crude EtOAc extract of *Penicillium* sp. HJT-A-6 was subjected to a full-scan HPLC-Q-TOF-MS/MS analysis. The obtained MS/MS data were used to generate the initial molecular network using the GNPS platform following the established protocol. This initial network was visualized and analyzed via Cytoscape 3.7.1 software.

**Figure 2:**
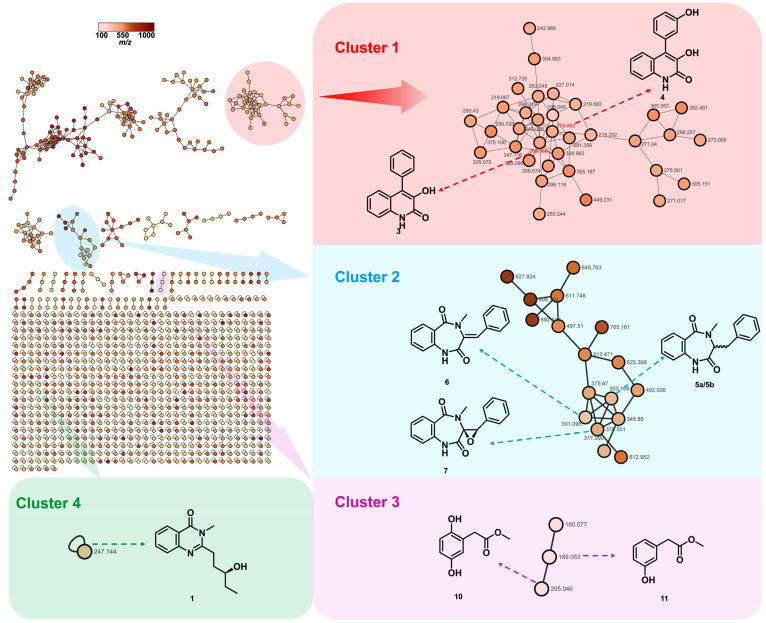
Molecular networking-guided chemometric discovery of peniquinazolinone A (**1**) and several known compounds (**3**–**7**, **9**, **10**).

As shown in Figure 2, the annotated nodes of the total secondary metabolites are displayed; the color of the node indicates the *m*/*z* of the parent ion. This process generated a network featuring 2373 nodes and 755 clusters; those representative highlighted clusters indicated several known natural products and a promising new compound. In cluster 1, highlighted in light red, the node *m*/*z* 276.062 was connected to the node *m*/*z* 260.069, which were supposed to be two known compounds, viridicatin (3) and viridicatol (4). Clusters 2 and 3, highlighted in light blue and light purple, respectively, were used to identify two classes of natural products, cyclopeptin analogues (5a/5b–7) and phenyl acetate derivates (10, 11). The orphan cluster 4 (*m*/*z* 247.144) in light green, which was not associated with any structurally known compound, indicates the presence of an unidentified compound. Thus, using *m*/*z* 247.144 as a guide, a new quinazolinone alkaloid, peniquinazolinone A (1), was isolated, along with known compounds 2–12.

### 2.2. Structure Elucidation of the Isolated Compounds

Compound **1** was obtained as a yellow oil. Its molecular formula was determined as C_14_H_18_N_2_O_2_ based on HRESIMS at *m*/*z* 269.1271 [M + Na]^+^ (calcd for 269.1266) (Figure S1), indicating seven degrees of unsaturation. The ^1^H NMR spectrum (Table 1 and Figure S3) revealed the resonances of four aromatic protons at δ_H_ 8.10 (d, J = 7.5 Hz, H-5), 7.77 (t, J = 7.5 Hz, H-3), 7.59 (d, J = 7.5 Hz, H-2), and 7.47 (d, J = 7.5 Hz, H-4), which indicated the presence of one 1,2-disubstituted phenyl group in **1**; three methylene protons at δ_H_ 1.43–2.99; one oxygenated methine proton at δ_H_ 3.48 (m, H-13); and two methyl protons at δ_H_ 3.56 (s) and 0.90 (t, J = 7.5 Hz, H-15). The ^13^C NMR and HSQC spectra (Table 1 and Figures S6 and S7) of **1** displayed the presence of fourteen carbons, including one carbonyl carbon, seven aromatic/olefinic carbons, three methylenes, one oxygenated methine, and two methyls.

**Figure 3:**
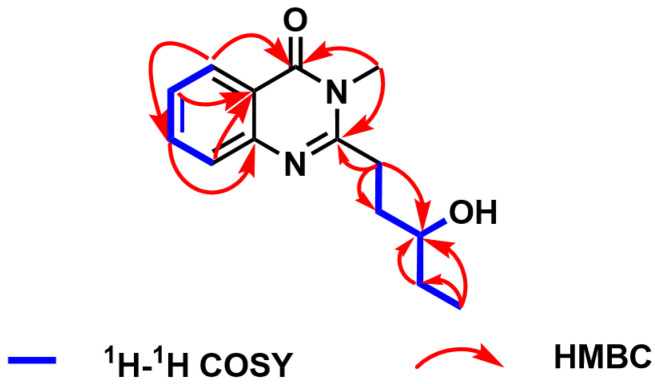
Key ^1^H-^1^H COSY and HMBC correlations of **1**.

The planar structure of compound **1** was deduced by HMBC and ^1^H–^1^H COSY spectra (Figure 3 and Figures S8 and S9). The HMBC correlations from H-2 to C-6, from H-3 to C-1, from H-4 to C-6, from H-5 to C-3 and C-7, and from H-16 to C-7 and C-9 established the structural skeleton of quinazolinone. The proton spin systems of H-11/H-12/H-13/H-14/H-15 and H-13/13-OH, coupled with the HMBC correlations from H-11 to C-9, C-12, and C-13; from H-14 to C-13; and from H-15 to C-13 and C-14, confirmed the linkage of the quinazolinone group and the pentan-3-ol moiety at C-9. Consequently, the planar structure of compound **1** was constructed, named peniquinazolinone A.

**Figure 4:**
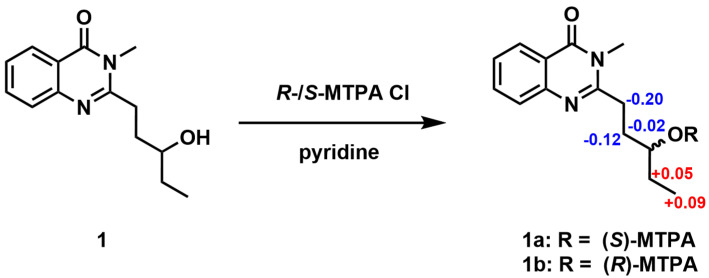
∆δ(δ_S_−δ_R_) values for MTPA esters of **1**.

The absolute configuration of C-13 was defined by the application of Mosher’s method. Compound **1** was reacted with both (R)-(–) and S-(+)-α-methoxy-α-(trifluoromethyl) phenylacetyl chlorides (MTPA-Cl) to afford the corresponding (S)- and (R)-Mosher esters (**1a** and **1b**), respectively. The absolute configuration at C-13 in **1** was determined to be R by the observed chemical shift differences, Δδ(δ_S_-δ_R_) (Figure 4 and Figures S4 and S5).

The known compounds, 2-(2-hydroxy-3-phenylpropionamido)-N-methylbenzamide (**2**) [26], viridicatin (**3**) [27], viridicatol (**4**) [27], (±)-cyclopeptin (**5a**/**5b**) [28,29], dehydrocyclopeptin (**6**) [29], cyclopenin (**7**) [30], cyclopenol (**8**) [31], methyl-indole-3-carboxylate (**9**) [32], 2,5-dihydroxyphenyl acetate (**10**) [33], methyl m-hydroxyphenylacetate (**11**) [34], and conidiogenone B (**12**) [35], were identified based on their ^1^H NMR and ^13^C NMR spectra (Figures S12–S32) and compared with those reported in the previous literature.

### 2.3. Seed-Germination-Promoting Activity of the Isolated Compounds

**Figure 5:**
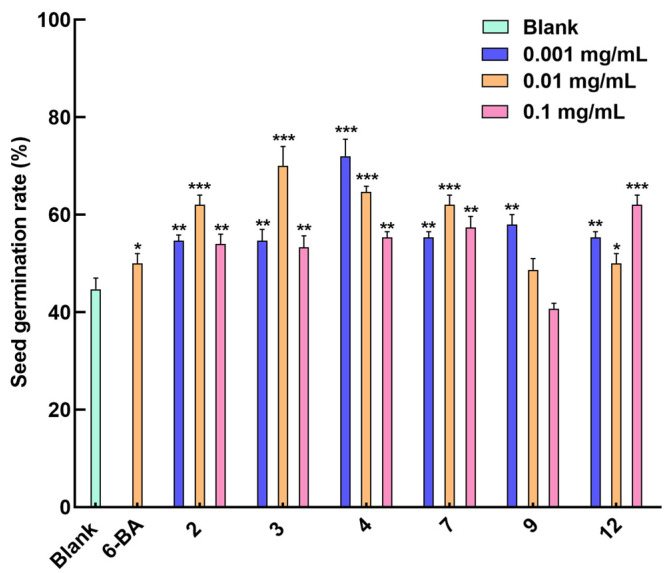
*Rhodiola tibetica* seed germination rate of **2**, **3**, **4**, **7**, **9**, and **12**. Columns represent the mean ± SD; *n* = 3; *** *p* < 0.001, ** *p* < 0.01, and * *p* < 0.05 vs. the blank control water.

**Figure 6:**
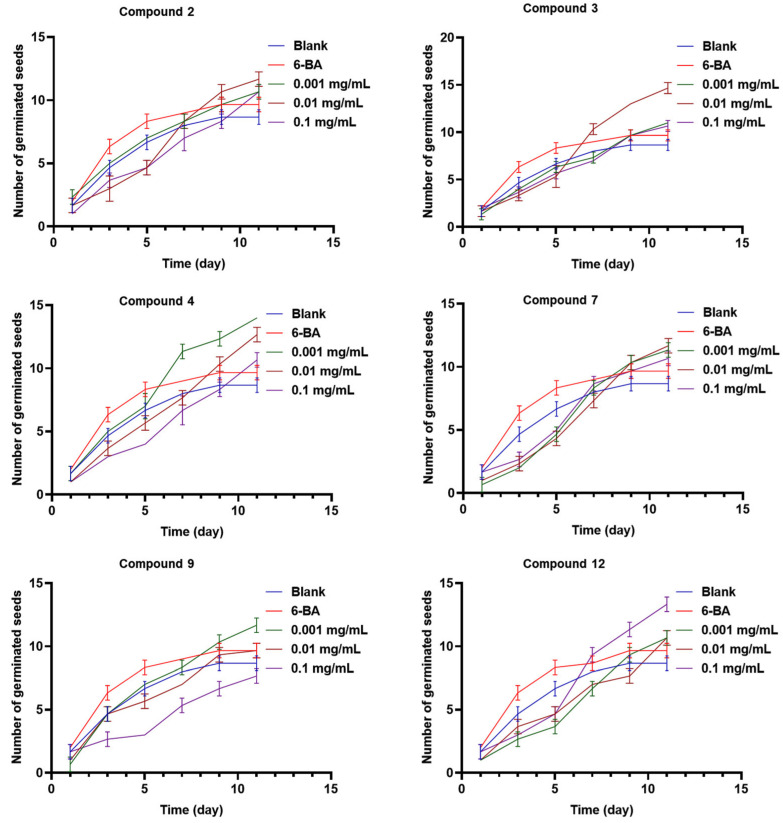
Effects of all the tested compounds, 6-BA, and the blank control water on the seed germination period of *Rhodiola tibetica*.

Compounds **2**, **3**, **4**, **7**, **9**, and **12** were tested for their seed-germination-promoting activity. As shown in Figure 5, compounds **2**, **3**, and **7** showed an optimal promoting effect on the seed germination of *Rhodiola tibetica* at a concentration of 0.01 mg/mL, with a germination rate of about 62%, 70%, and 62%, respectively, compared to the germination rate of about 52% of the positive drug 6-BA. Compounds **4** and **9** exhibited a germination rate of about 72% and 58%, respectively, at the optimal concentration of 0.001 mg/mL, indicating a negative correlation between concentration and germination rate. Compared with other tested compounds, compound **12** showed the best germination rate of about 62% at the same concentration level (0.1 mg/mL). In addition, compounds **2**, **3**, **4**, **7**, **9**, and **12** could delay the seed germination of *Rhodiola tibetica* up to the 11th day, while the positive drug 6-BA only affected the seed germination process until the 9th day (Figure 6). Compounds **2**, **3**, **7**, and **12** did not show concentration-dependent activity, which probably activated the upregulation of certain genes responsible for seed germination at concentrations ranging from 0.001 to 0.01 mg/mL. When the concentration exceeded a certain level, the expression levels of the genes responsible for seed germination decreased, hence not exhibiting concentration dependence. Additionally, the expression levels of certain genes responsible for seed germination were also influenced by the duration of time. Compound **12**, however, exhibited the opposite behavior.

Compound **1** was not tested for its seed-germination-promoting activity due to the trace amount. Compound **2** is a structural analogue of compound **1**, generated by the ring opening of the quinazoline moiety of compound **1**; further seed germination assays of compound **1** may verify whether the existence of the quinazoline moiety affects its seed-germination-promoting activity.

## 3. Materials and Methods

### 3.1. General Experimental Procedures

The UV spectrum was recorded on a Jasco V-560 spectrophotometer (JASCO Corporation, Kyoto, Japan). Optical rotation was obtained on an Autopol IV Polarimeter (Rudolph Research Analytical, Flanders, NJ, USA). CD spectrum was acquired on a Jasco J-810-150S spectropolarimeter (JASCO Corporation, Japan). High-resolution electrospray ionization mass spectrometry (HRESIMS) data were collected on an AB Sciex Triple TOF 4600 mass spectrometer (AB SCIEX, Framingham, MA, USA). NMR spectra were recorded on a Bruker Avance II 500 MHz NMR spectrometer (Bruker, Karlsruhe, Germany) with tetramethylsilane (TMS) as an internal standard. Agilent 1260 Infinity (Agilent Technologies Inc., Santa Clara, CA, USA), Waters 2535 (Waters Corporation, Milford, MA, USA), and Shimadzu LC-20AR (Shimadzu Corporation, Kyoto, Japan) semi-preparative HPLC systems were created using a Welch Ultimate XB-C18 column (250 mm × 10.0 mm, 5 μm). Silica gel (100−200 mesh and 200−300 mesh, Qingdao Marine Chemical Ltd., Qingdao, China) and Sephadex LH-20 (GE Healthcare Bio-Sciences AB, Uppsala, Sweden) were used for column chromatography. The silica gel GF254 (Qingdao Marine Chemical Co., Ltd., Qingdao, China) was used for analytical and preparative thin-layer chromatography (TLC).

### 3.2. Fungal Material

The fungus strain *Penicillium* sp. HJT-A-6 was obtained from the stem of *Rhodiola tibetica* collected in Langkazi County, Shannan City, Tibet, China, in July 2021. It was identified based on its morphological characteristics and its sequence in the internal transcribed spacer (ITS) analysis of rDNA, and the BLAST search result showed that the sequence was the most similar (99%) to the sequence of *Penicillium* sp. (compared to MN634462.1). The sequence data of the fungus were submitted to the GenBank database, accession number: OR346333.1. The fungus was deposited in the College of Life and Health, Dalian University, Dalian, China.

### 3.3. Fermentation and Isolation

The fungal strain was cultured on an autoclaved rice medium (one hundred 500 mL Erlenmeyer flasks, each containing 80 g rice, 110 mL water) in the stationary phase at 28 °C for 40 days. After 40 days, the fermentation material was cut into small pieces and extracted with 95% EtOH three times. The extract was concentrated under reduced pressure to afford an aqueous solution and then partitioned with petroleum ether, EtOAc and *n*-BuOH to obtain the EtOAc-soluble extract (64 g). The extract was subjected to silica gel column chromatography with CH_2_Cl_2_/MeOH (100:0–0:100) to afford fourteen fractions (Fr. A–N).

Fr. B (10.5 g) was chromatographed on a silica gel column with gradient elution (PE/EtOAc, 5:1–1:2) to yield 8 subfractions (Fr. B1–Fr. B8). Fr. B5 was further purified by semi-preparative HPLC with MeOH/H_2_O (40:60, 0–35 min, 3 mL/min) to obtain compound **11** (1.6 mg, *t*_R_ = 29 min).

Fr. C (6.3 g) was eluted with gradient petroleum (PE/EtOAc, 5:1–1:1) to afford 9 subfractions (Fr. C1–Fr. C9). Fr. C1 and Fr. C4 were subjected to the Sephadex LH-20 gel column and preparative TLC, respectively, to obtain compounds **3** (250 mg) and **12** (4.6 mg). Fr. C5 was chromatographed by the Sephadex LH-20 gel column using isocratic elution with CH_2_Cl_2_/MeOH (1:1), yielding Fr. C5b, which was purified by semi-preparative HPLC with CH_3_CN/H_2_O (30:70, 0–30 min, 3 mL/min) to give rise to compound **6** (13.6 mg, *t_R_* = 22 min). Fr. C7 was purified by semi-preparative HPLC with MeOH/H_2_O (60:40, 0–40 min, 3 mL/min) to obtain compound **7** (193 mg, *t*_R_ = 14 min) and a mixture of **5a** and **5b** in a 1:1.7 molar ratio (13.8 mg, *t*_R_ = 17 min). Fr. C8 was applied to the Sephadex LH-20 gel column with CH_2_Cl_2_/MeOH (1:1) to give rise to Fr. C8e, which was purified by semi-preparative HPLC with the gradient MeOH/H_2_O (20:80–60:40, 0–30 min, 3 mL/min) to obtain compound **9** (20 mg, *t*_R_ = 25 min).

Fr. E (6.3 g) was chromatographed on a silica gel column with gradient elution (PE/EtOAc, 7:1–1:2), affording 20 fractions (Fr. E1–Fr. E20). Fr. E13 was purified by semi-preparative HPLC with the gradient MeOH/H_2_O (20:80–95:5, 0–40 min, 3 mL/min) to yield compound **1** (1.6 mg, *t*_R_ = 26 min). Fr. E14 was purified by semi-preparative HPLC with MeOH/H_2_O (40:60, 0–40 min, 3 mL/min) to obtain compound **2** (3.4 mg, *t*_R_ = 16 min) and compound **4** (120 mg, *t*_R_ = 32 min). Fr. E16 was purified by semi-preparative HPLC with CH_3_CN/H_2_O (45:55, 0–25 min, 3 mL/min) to afford compound **8** (6 mg, *t*_R_ = 12 min).

Fr. G (6.7g) was chromatographed on a silica gel column with gradient elution (PE/EtOAc, 10:1–1:1) to afford 5 subfractions (Fr. G1–Fr. G5). Fr. G4 was applied to the Sephadex LH-20 gel column with CH_2_Cl_2_/MeOH (1:1) to yield Fr. G4d, which was purified by semi-preparative HPLC with the gradient MeOH/H_2_O (20:80–95:5, 0–30 min, 3 mL/min) to obtain compound **10** (5.8 mg, *t*_R_ = 10 min).

Peniquinazolinone A (**1**): yellow oil; ${\left[\alpha \right]}_{D}^{20}$ +20 (*c* 0.2, MeOH); UV (CH_3_OH) *λ*_max_ (log *ε*) 223 (4.24), 267 (3.74) nm; ECD (CH_3_OH) *λ*_max_ (Δ*ε*) 220 (–7.0) nm; ^1^H NMR (DMSO-*d*_6_, 500 MHz); and ^13^C NMR (DMSO-*d*_6_, 125 MHz) data (see Table 1); HRESIMS *m*/*z* 269.1271 [M + Na]^+^ (calculated for C_14_H_18_N_2_O_2_Na, 269.1266).

### 3.4. Mosher Esterification of Compound ***1***

Compound **1** (0.5 mg) was dissolved in 100 μL of CDCl_3_ in an NMR tube, and sequentially, 9 μL of pyridine and 15 μL of (*R*)-(–)-α-methoxy-α-(trifluoromethyl)phenylacetyl chloride ((*R*)-(–)-MTPA-Cl) were added. The mixture was stirred at room temperature for 1 h to afford the corresponding (*S*)-Mosher ester (**1a**) and subsequently diluted with 300 μL of CDCl_3_ to acquire a ^1^H NMR spectrum. The (*R*)-Mosher ester (**1b**) of **1** was prepared from (*S*)-(+)-MTPA-Cl using the same method.

### 3.5. Seed-Germination-Promoting Assay

Compounds **2**, **3**, **4**, **7**, **9**, and **12** were dissolved in a 0.2% DMSO aqueous solution to yield a stock solution with a concentration of 0.06 mg/mL. Then, 5 µL, 50 µL, and 500 µL of compounds **2**, **3**, **4**, **7**, **9**, and **12** were added to a 30 mm filter paper placed in a 6-well plate. After the evaporation of the solvent, the filter paper was immersed in 300 µL of distilled water, and then 20 seeds of *Rhodiola tibetica* were displayed in each 30 mm filter paper and incubated with a light–dark regime of 16:8 h at 20 °C for 7 days. The germination rate of the seeds was calculated after incubation. The experimental data were collected from three independent experiments. Further experiments on the relationship between the number of germinated seeds and germination time for compounds **2**, **3**, **4**, **7**, **9**, and **12** were also conducted; the germination period was set up to 11 days.

## 4. Conclusions

In this work, the chemical investigation of the *Rhodiola tibetica* endophytic fungus *Penicillium* sp. HJT-A-6 led to the isolation and identification of a new quinazolinone alkaloid named peniquinazolinone A (**1**) and eleven known compounds. Compound **1** was identified through an extensive spectroscopic analysis and the modified Mosher’s method. Compounds **2**, **3**, and **7** showed an optimal promoting effect on the seed germination of *Rhodiola tibetica* at a concentration of 0.01 mg/mL. Compounds **4** and **9** had optimal seed-germination-promoting activity at a concentration of 0.001 mg/mL, while the optimal concentration for compound **12** to promote *Rhodiola tibetica* seed germination was 0.1 mg/mL. Additionally, all the tested compounds assuredly delayed the seed germination of the host plant. Furthermore, the mechanism of these known compounds with seed-germination-promoting activity will be investigated using biochemical and transcriptomic methods. These above results not only broadened the structural diversity of quinazoline metabolites derived from fungi but also provided data support for understanding the interactive relationship between endophytic fungi and host plants.

## Figures and Tables

**Table 1:** ^1^H and ^13^C NMR data for compound **1** in DMSO-*d*_6_.

Position	*δ*_H_ (mult., *J* in Hz)	*δ*_C_, Type
1	—	147.4, C
2	7.59 (d, 7.5)	127.1, C
3	7.77 (t, 7.5)	134.6, C
4	7.47 (t, 7.5)	126.6, C
5	8.10 (d, 7.5)	126.6, C
6	—	120.2, C
7	—	161.9, C
8	—	—
9	—	158.6, C
10	—	—
11	2.99 (m)	31.6, CH_2_
	2.85 (m)	
12	1.92 (m)	33.7, CH_2_
	1.73 (m)	
13	3.48 (m)	71.0, CH
14	1.43 (m)	30.4, CH_2_
15	0.90 (t, 7.5)	10.6, CH_3_
16	3.56 (s)	30.4, CH_3_
13-OH	4.57 (s)	**—**

## Data Availability

Data are contained within the article and Supplementary Materials.

## Supplementary Materials

The following supporting information can be downloaded at https://www.mdpi.com/article/10.3390/molecules29092112/s1: Figure S1: Positive-mode HRESIMS spectrum of **1**; Figure S2: UV spectrum of **1**; Figure S3: ^1^H NMR (DMSO-*d*_6_, 500 MHz) spectrum of **1**; Figure S4: ^1^H NMR (DMSO-*d*_6_, 500 MHz) spectrum of **1a**; Figure S5: ^1^H NMR (DMSO-*d*_6_, 500 MHz) spectrum of **1b**; Figure S6: ^13^C NMR (DMSO-*d*_6_, 125 MHz) spectrum of **1**; Figure S7: HSQC (DMSO-*d*_6_, 500 MHz) spectrum of **1**; Figure S8: HMBC (DMSO-*d*_6_, 500 MHz) spectrum of **1**; Figure S9: ^1^H-^1^H COSY (DMSO-*d*_6_, 500 MHz) spectrum of **1**; Figure S10: NOESY (DMSO-*d*_6_, 500 MHz) spectrum of **1**; Figure S11: CD spectrum of **1**; Figure S12: ^1^H NMR (DMSO-*d*_6_, 500 MHz) spectrum of **2**; Figure S13: ^13^C NMR (DMSO-*d*_6_, 125 MHz) spectrum of **2**; Figure S14: ^1^H NMR (DMSO-*d*_6_, 500 MHz) spectrum of **3**; Figure S15: ^13^C NMR (DMSO-*d*_6_, 125 MHz) spectrum of **3**; Figure S16: ^1^H NMR (DMSO-*d*_6_, 500 MHz) spectrum of **4**; Figure S17: ^13^C NMR (DMSO-*d*_6_, 125 MHz) spectrum of **4**; Figure S18: ^1^H NMR (DMSO-*d*_6_, 500 MHz) spectrum of **5a/5b**; Figure S19: ^13^C NMR (DMSO-*d*_6_, 125 MHz) spectrum of **5a/5b**; Figure S20: ^1^H NMR (DMSO-*d*_6_, 500 MHz) spectrum of **6**; Figure S21: ^13^C NMR (DMSO-*d*_6_, 125 MHz) spectrum of **6**; Figure S22: ^1^H NMR (DMSO-*d*_6_, 500 MHz) spectrum of **7**; Figure S23: ^13^C NMR (DMSO-*d*_6_, 125 MHz) spectrum of **7**; Figure S24: ^1^H NMR (DMSO-*d*_6_, 500 MHz) spectrum of **8**; Figure S25: ^13^C NMR (DMSO-*d*_6_, 125 MHz) spectrum of **8**; Figure S26: ^1^H NMR (DMSO-*d*_6_, 500 MHz) spectrum of **9**; Figure S27: ^1^H NMR (DMSO-*d*_6_, 500 MHz) spectrum of **10**; Figure S28: ^13^C NMR (DMSO-*d*_6_, 125 MHz) spectrum of **10**; Figure S29: ^1^H NMR (DMSO-*d*_6_, 500 MHz) spectrum of **11**; Figure S30: ^13^C NMR (DMSO-*d*_6_, 125 MHz) spectrum of **11**; Figure S31: ^1^H NMR (DMSO-*d*_6_, 500 MHz) spectrum of **12**; Figure S32: ^13^C NMR (DMSO-*d*_6_, 125 MHz) spectrum of **12**.
