# Supplementary material for: A New Quinazolinone Alkaloid along with Known Compounds with Seed-Germination-Promoting Activity from Rhodiola tibetica Endophytic Fungus Penicillium sp. HJT-A-6
Source: Molecules. 2024 May 2;29(9):2112. doi: 10.3390/molecules29092112 (PMC11085523; doi:10.3390/molecules29092112)
Supplement: Supplementary file 1 [file molecules-29-02112-s001.zip › molecules-2986028-supplementary.pdf]

Article

A New Quinazolinone Alkaloid along with Known Compounds with Seed-Germination-Promoting Activity from *Rhodiola tibetica* Endophytic Fungus *Penicillium* sp. HJT-A-6

Dongliang Xiao [1,†], Yan Wang [1,†], Congcong Gao [1], Xuemei Zhang [1], Weixing Feng [1], Xuan Lu [1,*], Baomin Feng [1,*]

1 College of life and health, Dalian University, Dalian 116622, China; xdl120318@163.com (D.X.); wangyan_9910@163.com (Y.W.); gcc1125@163.com (C.G.); zxuemei2024@163.com (X.Z.); fwx_0910@163.com (W.F.);

* Correspondence: luxuan_232@163.com (X.L.); fbmdlu@163.com (B.F.)

† These authors contributed equally to this work.

Supplementary Materials

Table of Contents

**Figure S1**. Positive mode HRESIMS spectrum of **1**	3
**Figure S2**. UV spectrum of **1**	3
**Figure S3**. ^{1}H NMR (DMSO-d_6, 500 MHz) spectrum of **1**	4
**Figure S4**. ^{1}H NMR (DMSO-d_6, 500 MHz) spectrum of **1**a	4
**Figure S5**. ^{1}H NMR (DMSO-d_6, 500 MHz) spectrum of **1**b	5
**Figure S6**. ^{13}C NMR (DMSO-d_6, 125 MHz) spectrum of **1**	5
**Figure S7**. HSQC (DMSO-d_6, 500 MHz) spectrum of **1**	6
**Figure S8**. HMBC (DMSO-d_6, 500 MHz) spectrum of **1**	6
**Figure S9**. ^{1}H-^{1}H COSY (DMSO-d_6, 500 MHz) spectrum of **1**	7
**Figure S10**. NOESY (DMSO-d_6, 500 MHz) spectrum of **1**	7
**Figure S11**. CD spectrum of **1**	8
**Figure S12**. ^{1}H NMR (DMSO-d_6, 500 MHz) spectrum of **2**	9
**Figure S13**. ^{13}C NMR (DMSO-d_6, 125 MHz) spectrum of **2**	9
**Figure S14**. ^{1}H NMR (DMSO-d_6, 500 MHz) spectrum of **3**	10
**Figure S15**. ^{13}C NMR (DMSO-d_6, 125 MHz) spectrum of **3**	10
**Figure S16**. ^{1}H NMR (DMSO-d_6, 500 MHz) spectrum of **4**	11
**Figure S17**. ^{13}C NMR (DMSO-d_6, 125 MHz) spectrum of **4**	11
**Figure S18**. ^{1}H NMR (DMSO-d_6, 500 MHz) spectrum of **5a/5b**	12
**Figure S19**. ^{13}C NMR (DMSO-d_6, 125 MHz) spectrum of **5a/5b**	12
**Figure S20**. ^{1}H NMR (DMSO-d_6, 500 MHz) spectrum of **6**	13
**Figure S21**. ^{13}C NMR (DMSO-d_6, 125 MHz) spectrum of **6**	13
**Figure S22**. ^{1}H NMR (DMSO-d_6, 500 MHz) spectrum of **7**	14
**Figure S23**. ^{13}C NMR (DMSO-d_6, 125 MHz) spectrum of **7**	14
**Figure S24**. ^{1}H NMR (DMSO-d_6, 500 MHz) spectrum of **8**	15
**Figure S25**. ^{13}C NMR (DMSO-d_6, 125 MHz) spectrum of **8**	15
**Figure S26**. ^{1}H NMR (DMSO-d_6, 500 MHz) spectrum of **9**	16
**Figure S27**. ^{1}H NMR (DMSO-d_6, 500 MHz) spectrum of **10**	17
**Figure S28**. ^{13}C NMR (DMSO-d_6, 125 MHz) spectrum of **10**	17
**Figure S29**. ^{1}H NMR (DMSO-d_6, 500 MHz) spectrum of **11**	18
**Figure S30**. ^{13}C NMR (DMSO-d_6, 125 MHz) spectrum of **11**	18
**Figure S31**. ^{1}H NMR (DMSO-d_6, 500 MHz) spectrum of **12**	19
**Figure S32**. ^{13}C NMR (DMSO-d_6, 125 MHz) spectrum of **12**	19

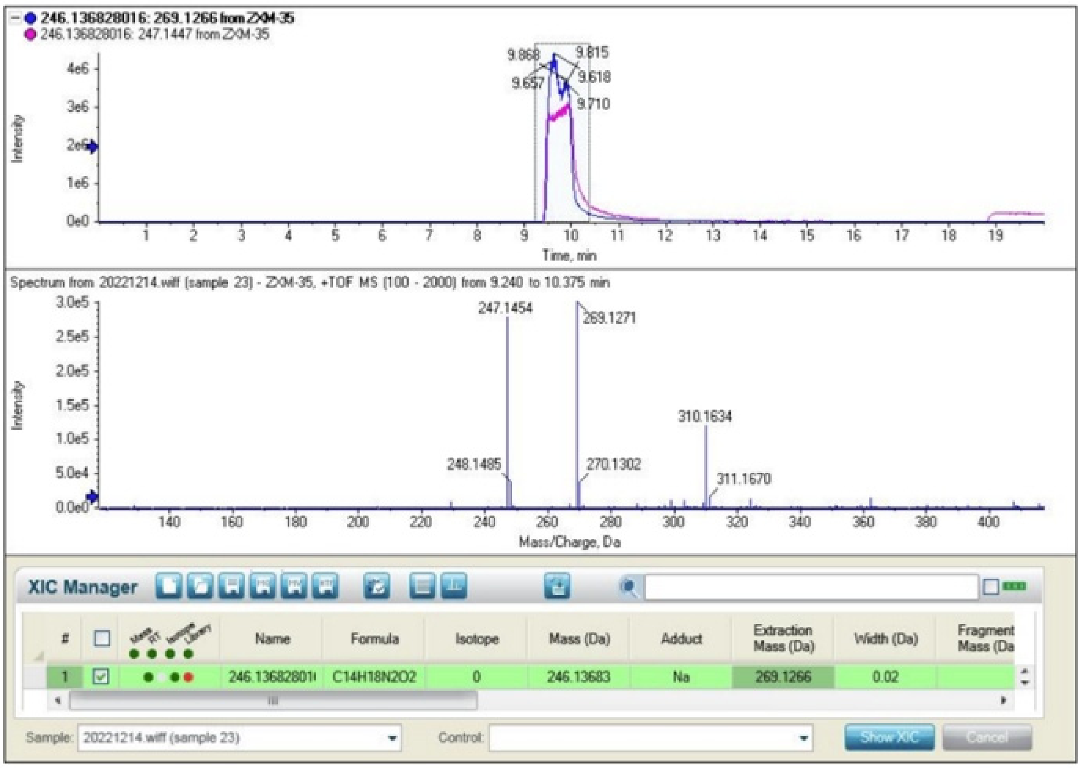

Figure S1. Positive mode HRESIMS spectrum of **1**

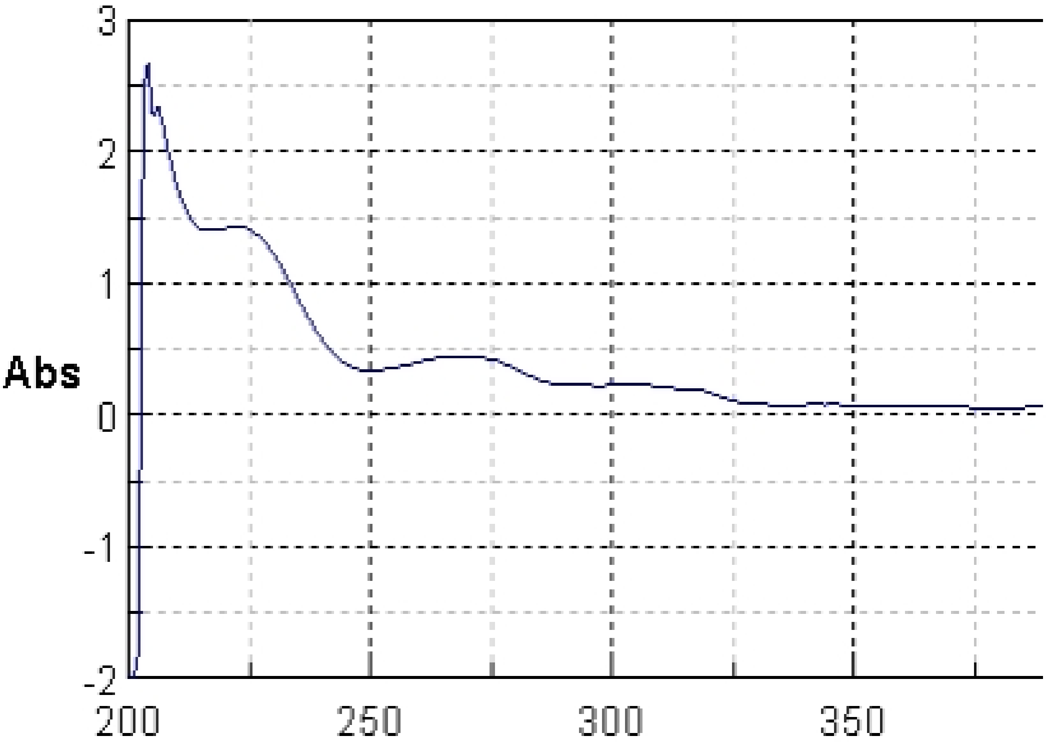

Figure S2. UV spectrum of **1**

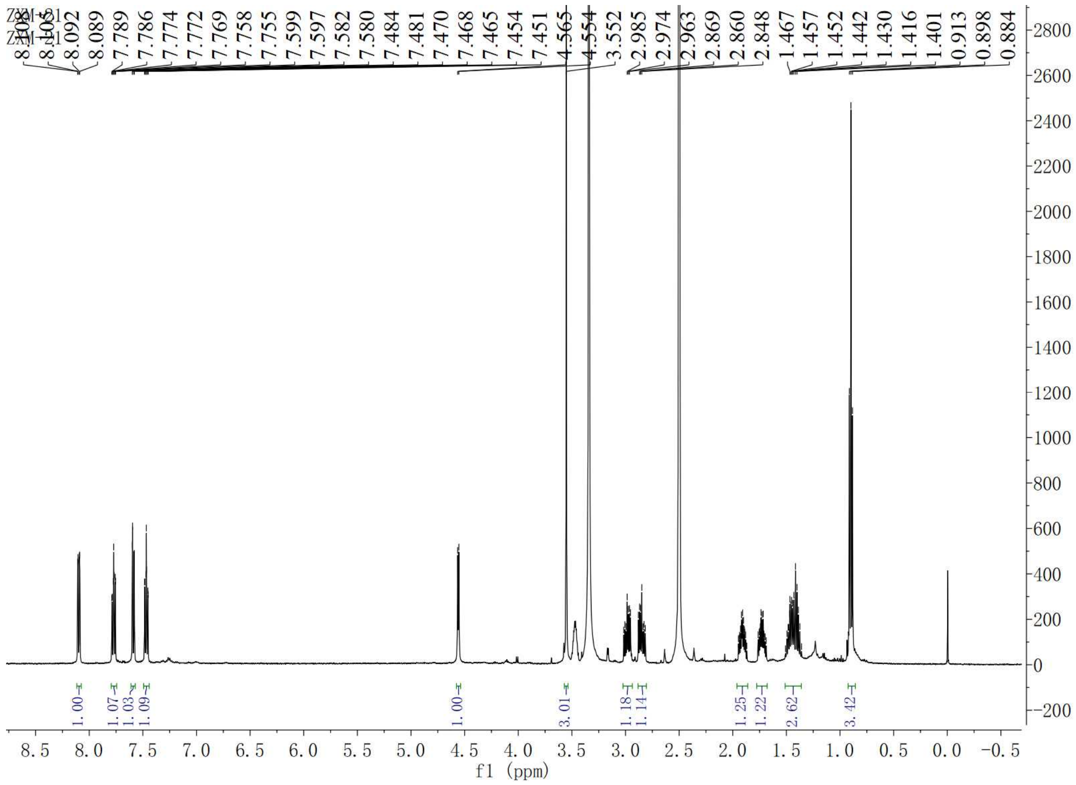

Figure S3. ^{1}H NMR (DMSO-d_6, 500 MHz) spectrum of **1**

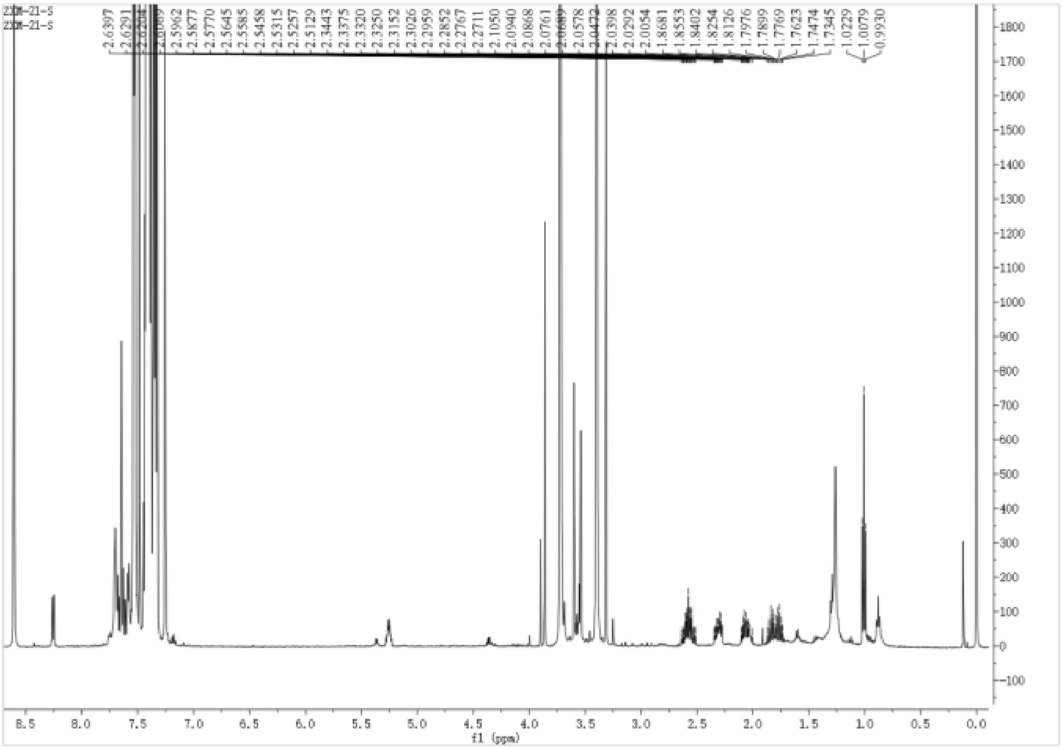

Figure S4. ^{1}H NMR (DMSO-d_6, 500 MHz) spectrum of **1a**

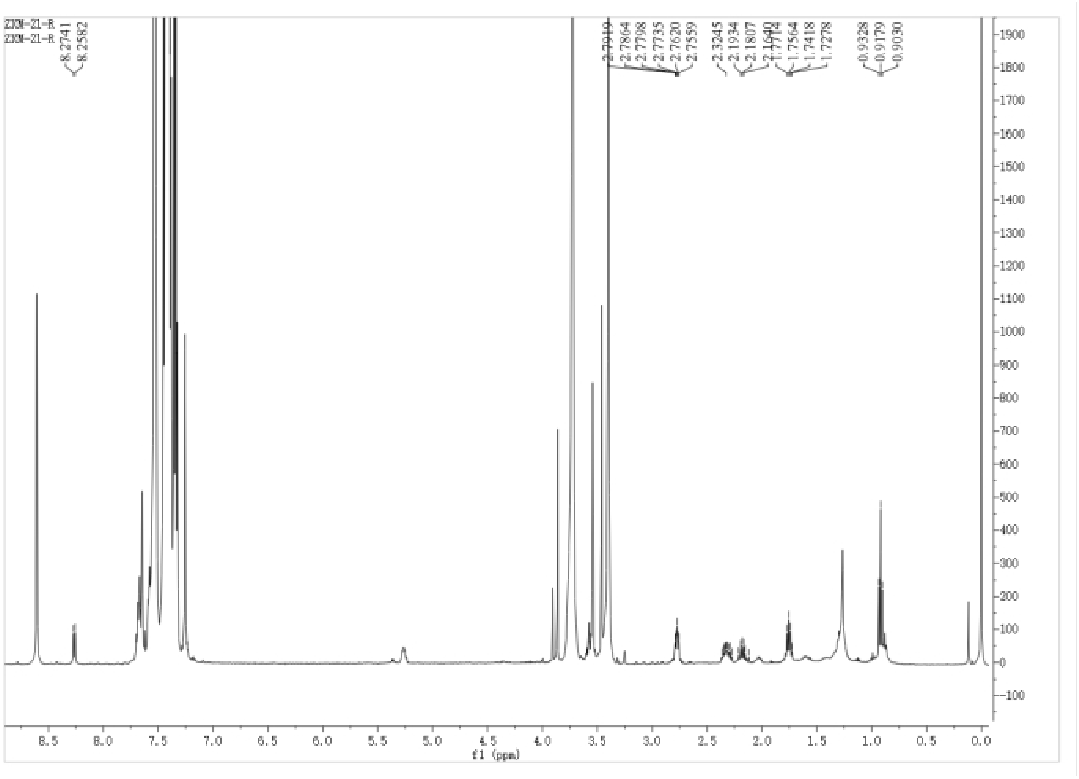

Figure S5. ^{1}H NMR (DMSO-d_6, 500 MHz) spectrum of **1b**

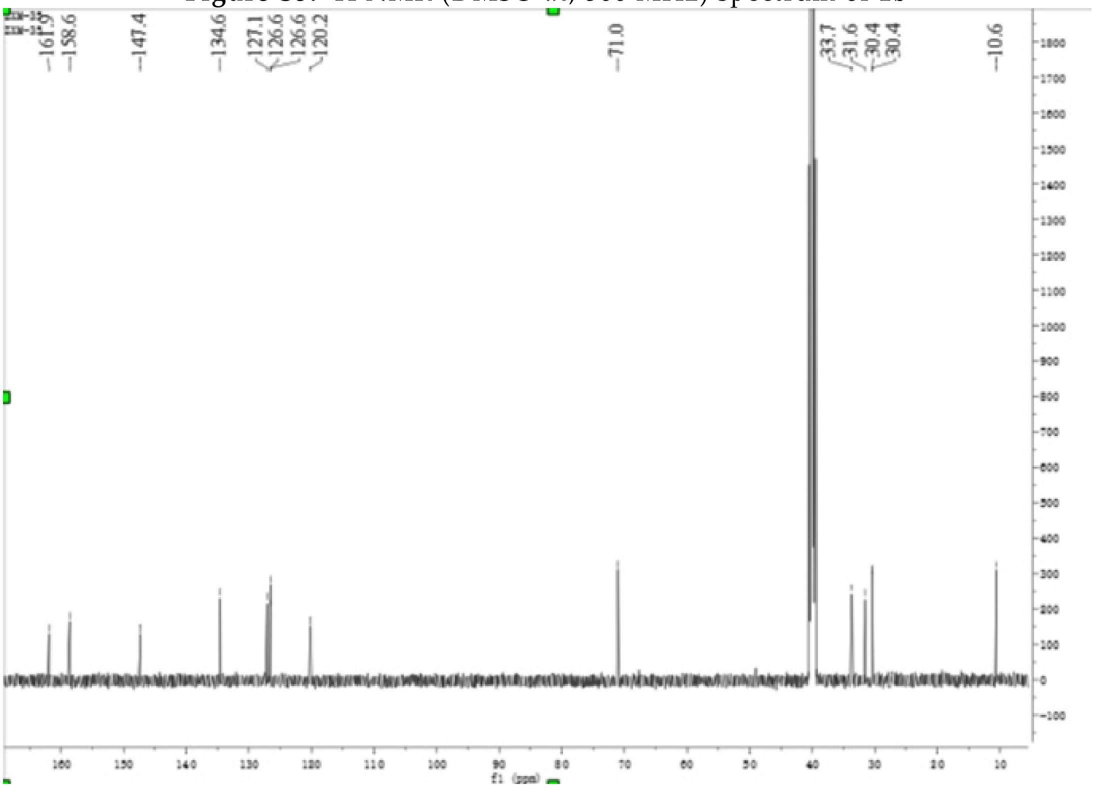

Figure S6. ^{13}C NMR (DMSO-d_6, 125 MHz) spectrum of **1**

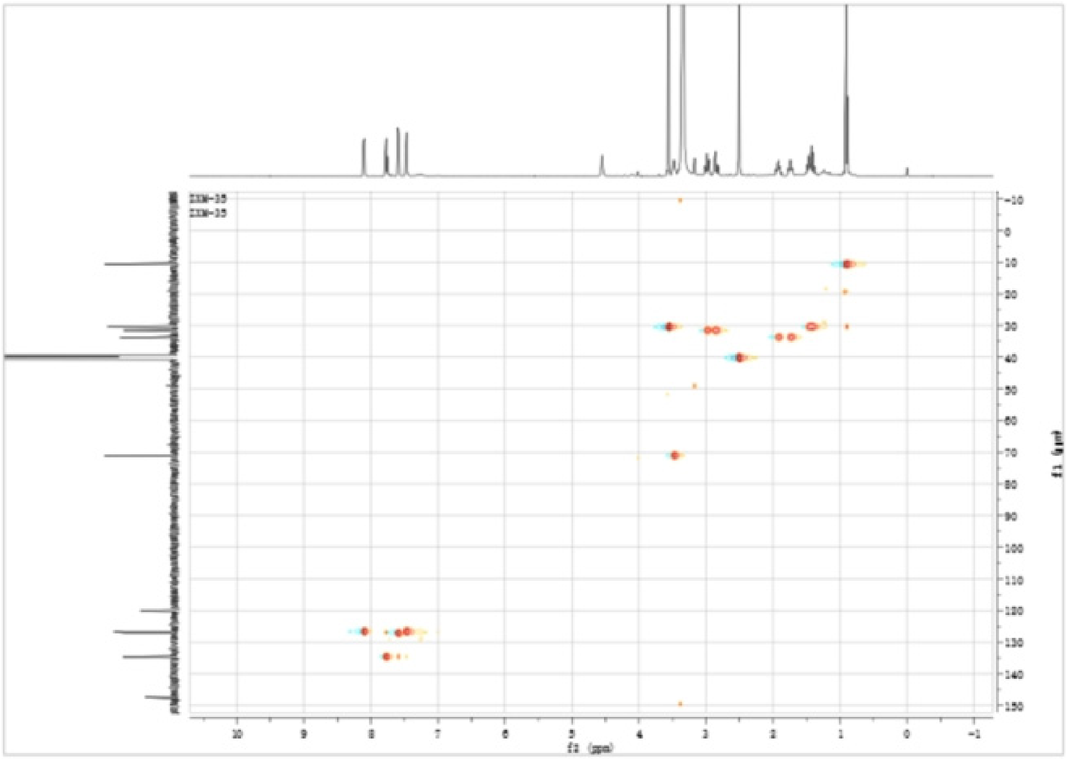

Figure S7. HSQC (DMSO-d_6, 500 MHz) spectrum of **1**

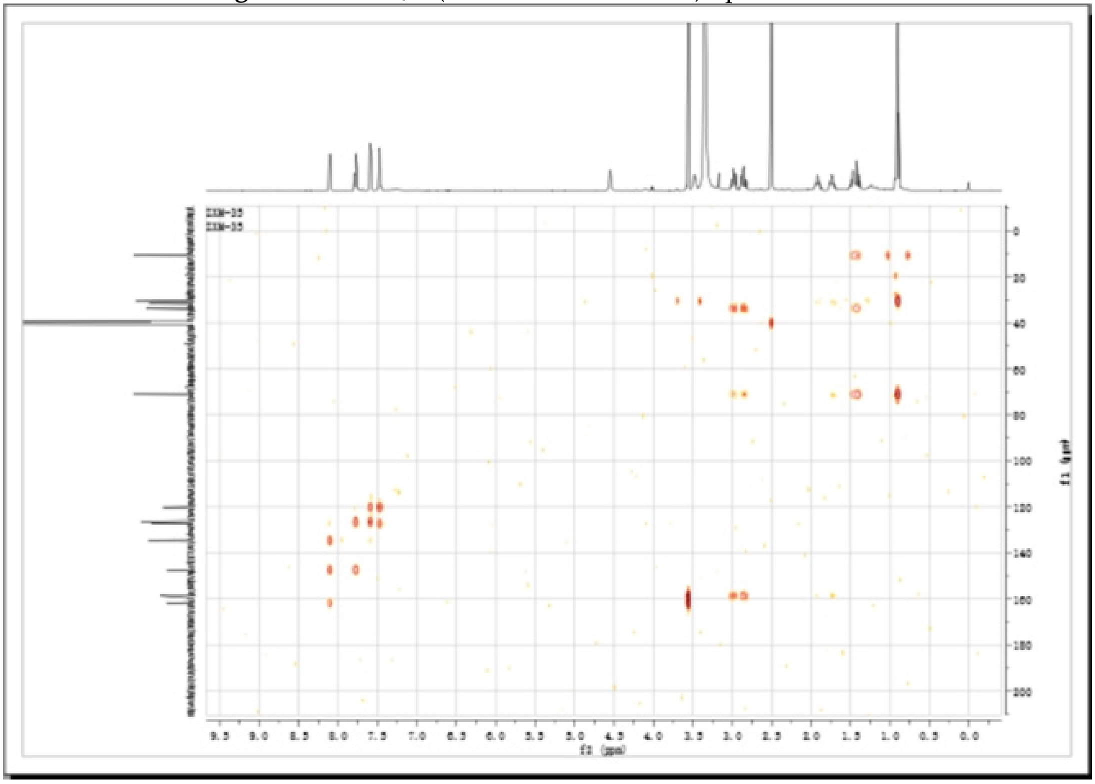

Figure S8. HMBC (DMSO-d_6, 500 MHz) spectrum of **1**

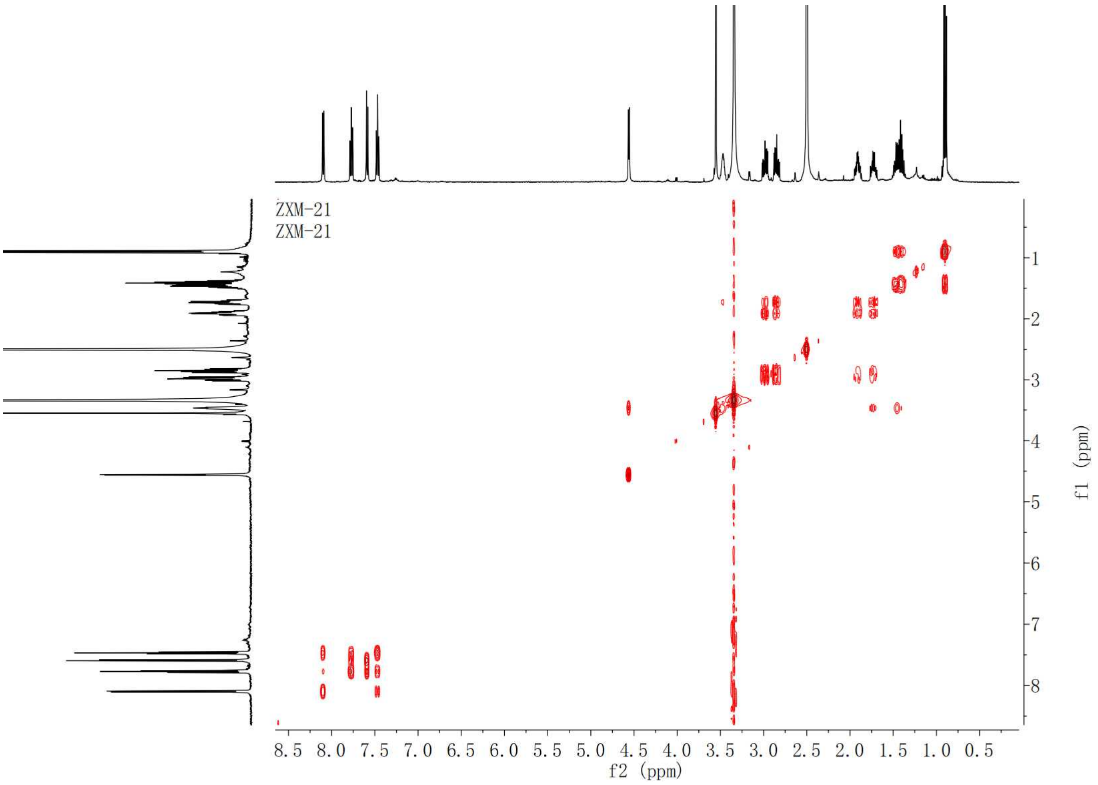

Figure S9. ^{1}H-^{1}H COSY (DMSO-d_6, 500 MHz) spectrum of **1**

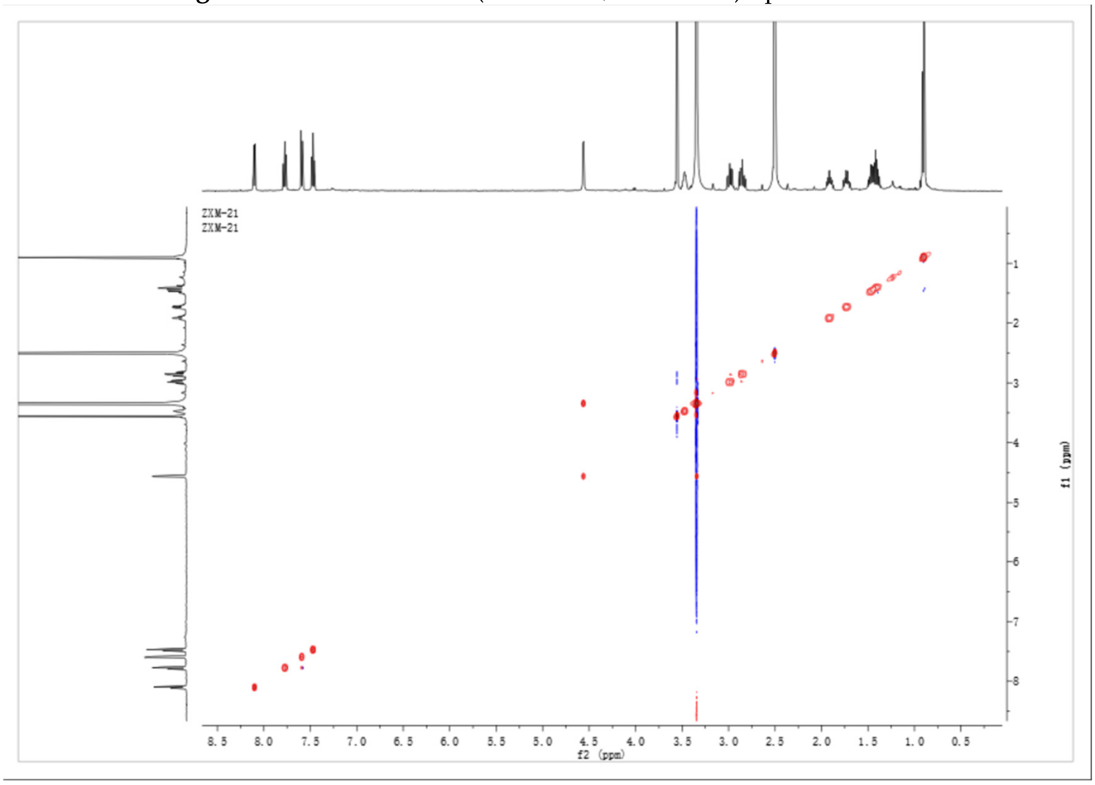

Figure S10. NOESY (DMSO-d_6, 500 MHz) spectrum of **1**

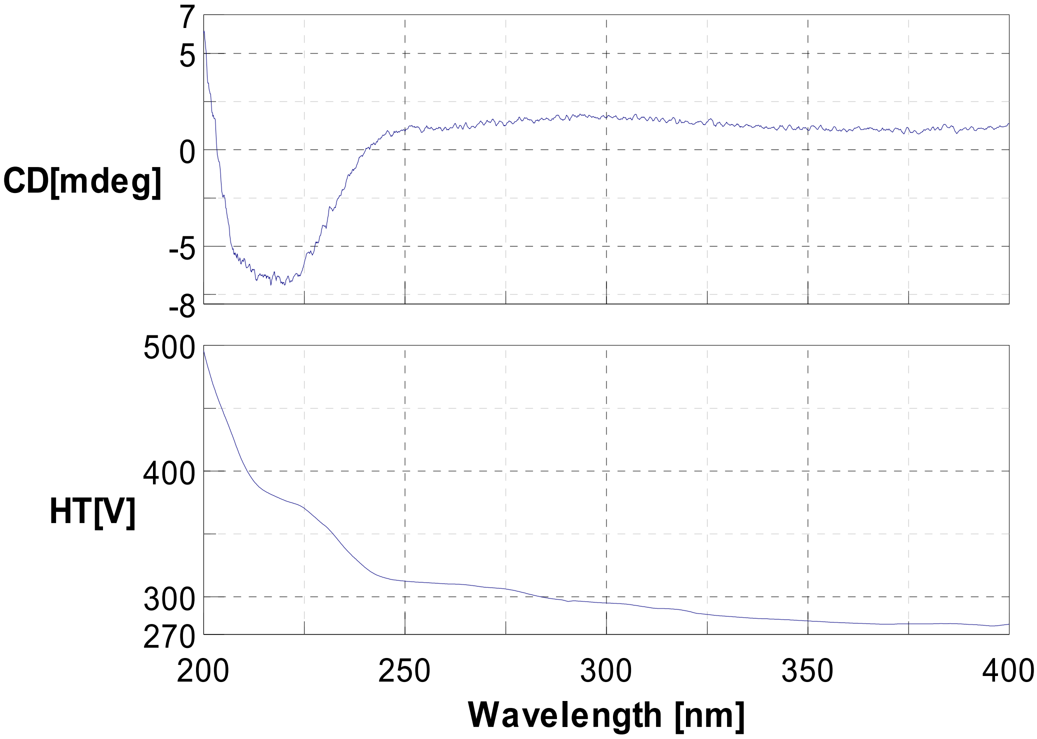

Figure S11. CD spectrum of **1**

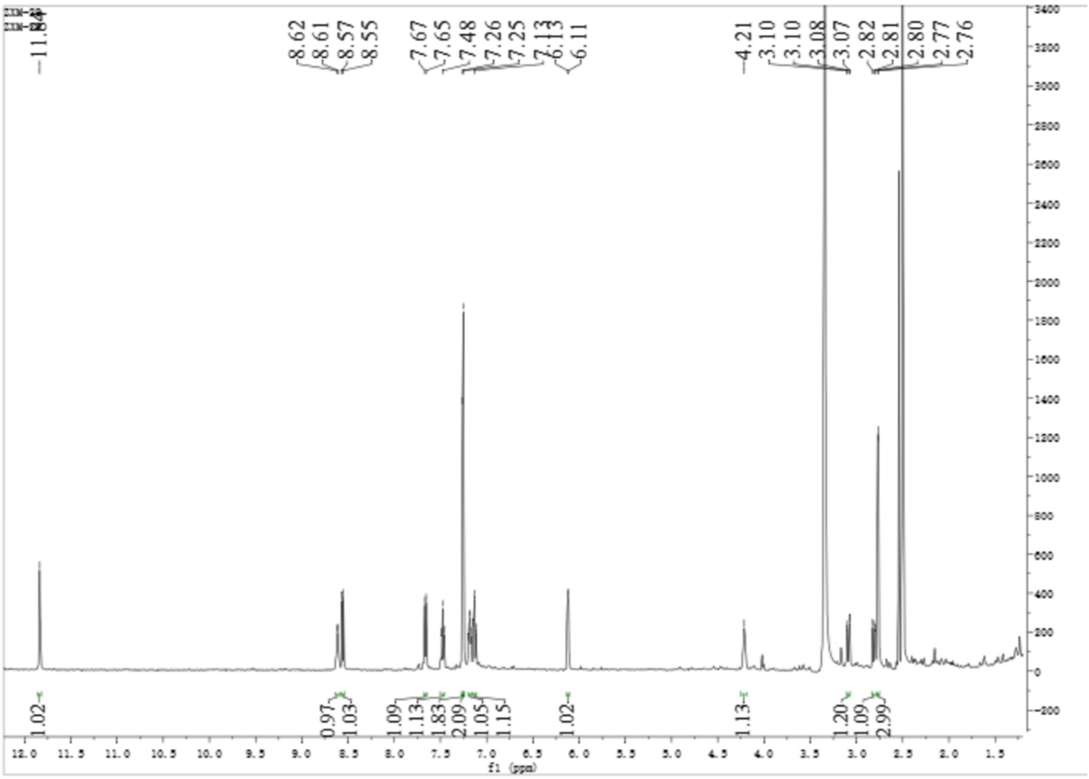

Figure S12. ^{1}H NMR (DMSO-d_6, 500 MHz) spectrum of **2**

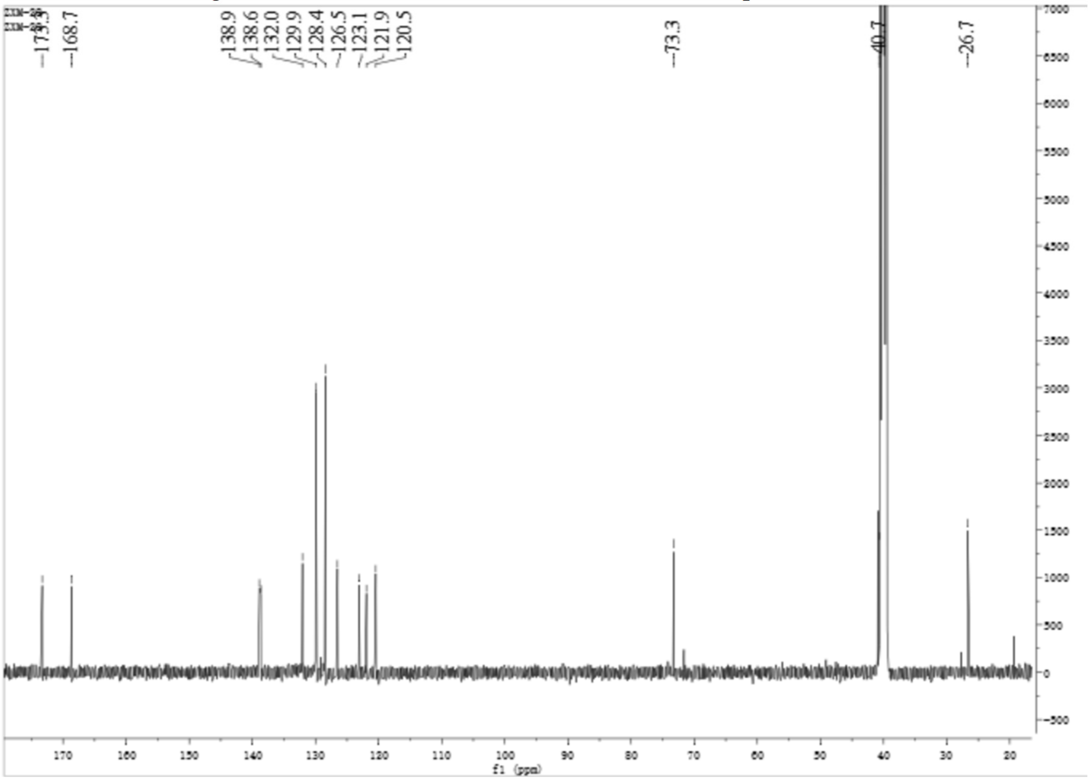

Figure S13. ^{13}C NMR (DMSO-d_6, 125 MHz) spectrum of **2**

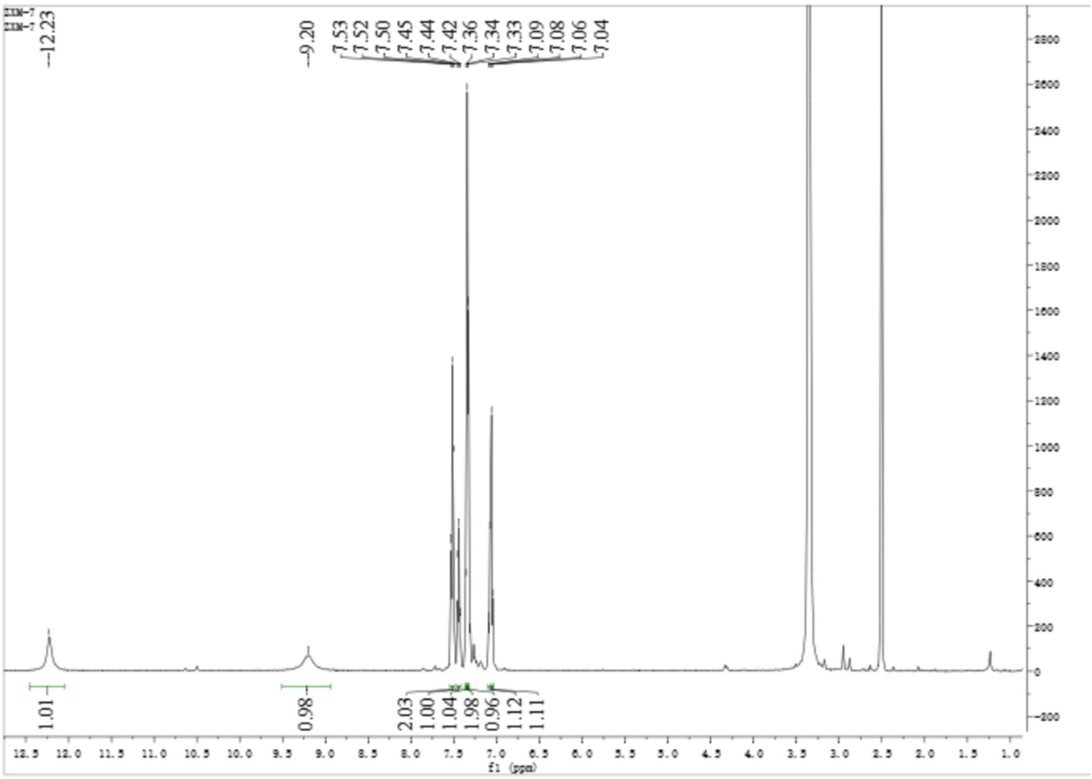

Figure S14. ^{1}H NMR (DMSO-d_6, 500 MHz) spectrum of **3**

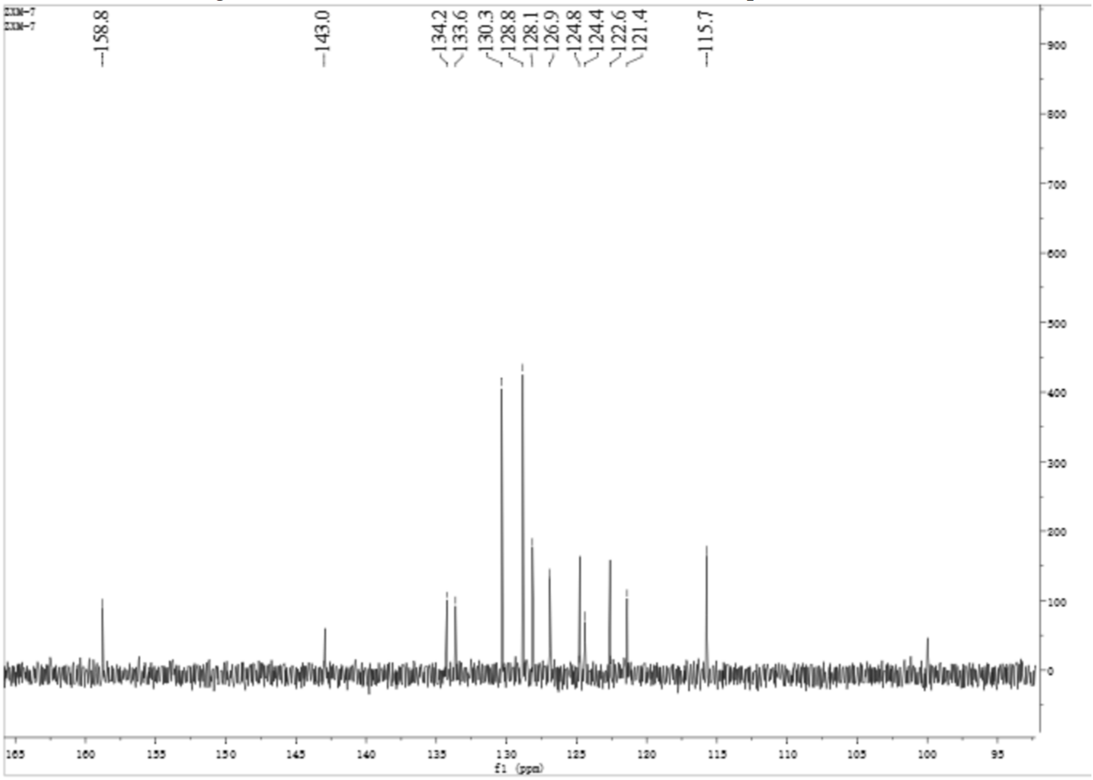

Figure S15. ^{13}C NMR (DMSO-d_6, 125 MHz) spectrum of **3**

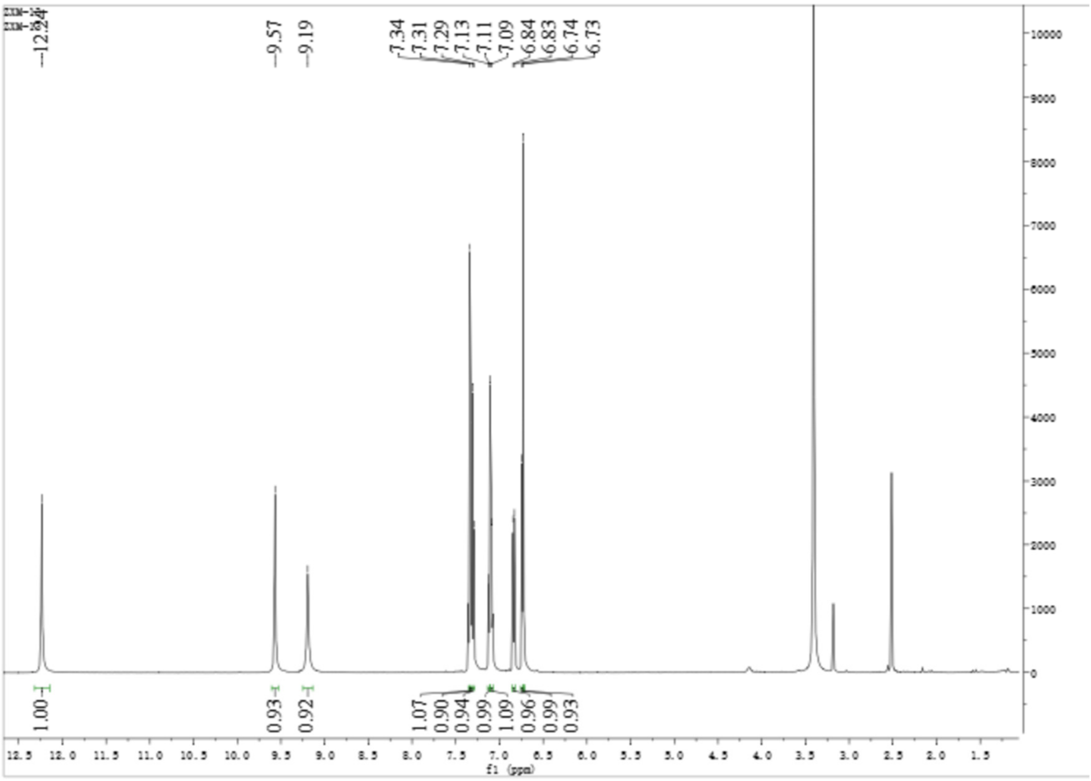

Figure S16. ^{1}H NMR (DMSO-d_6, 500 MHz) spectrum of **4**

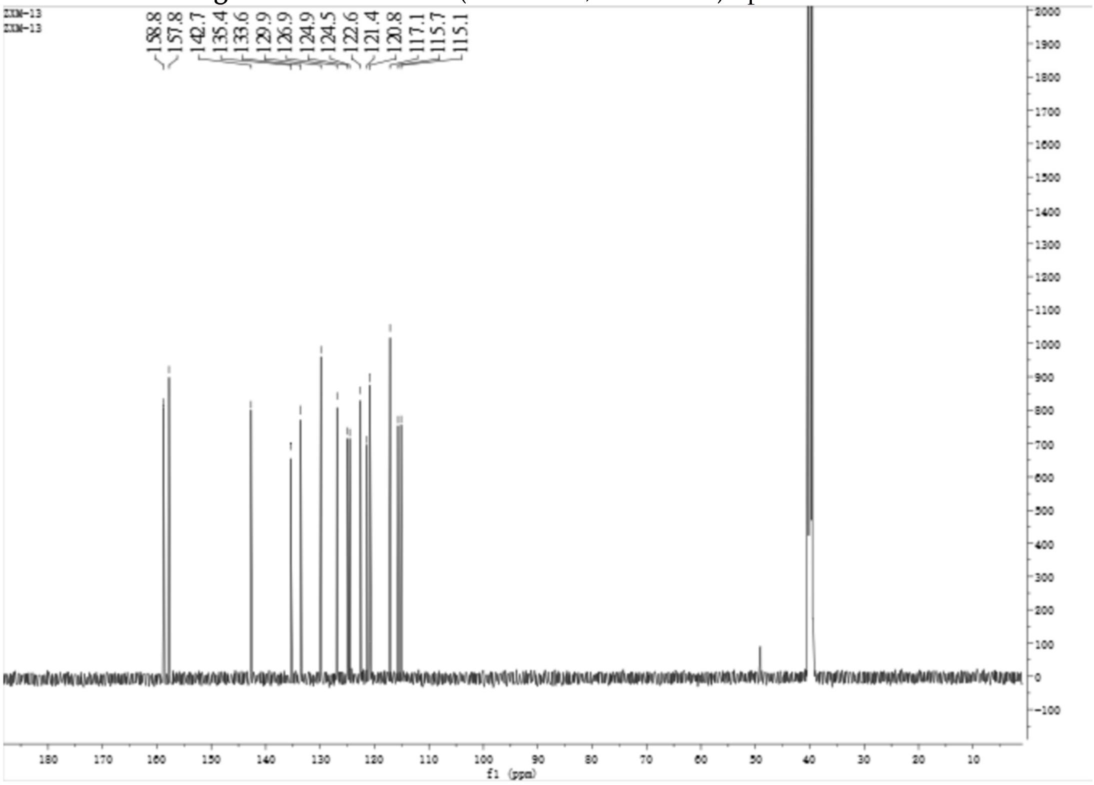

Figure S17. ^{13}C NMR (DMSO-d_6, 125 MHz) spectrum of **4**

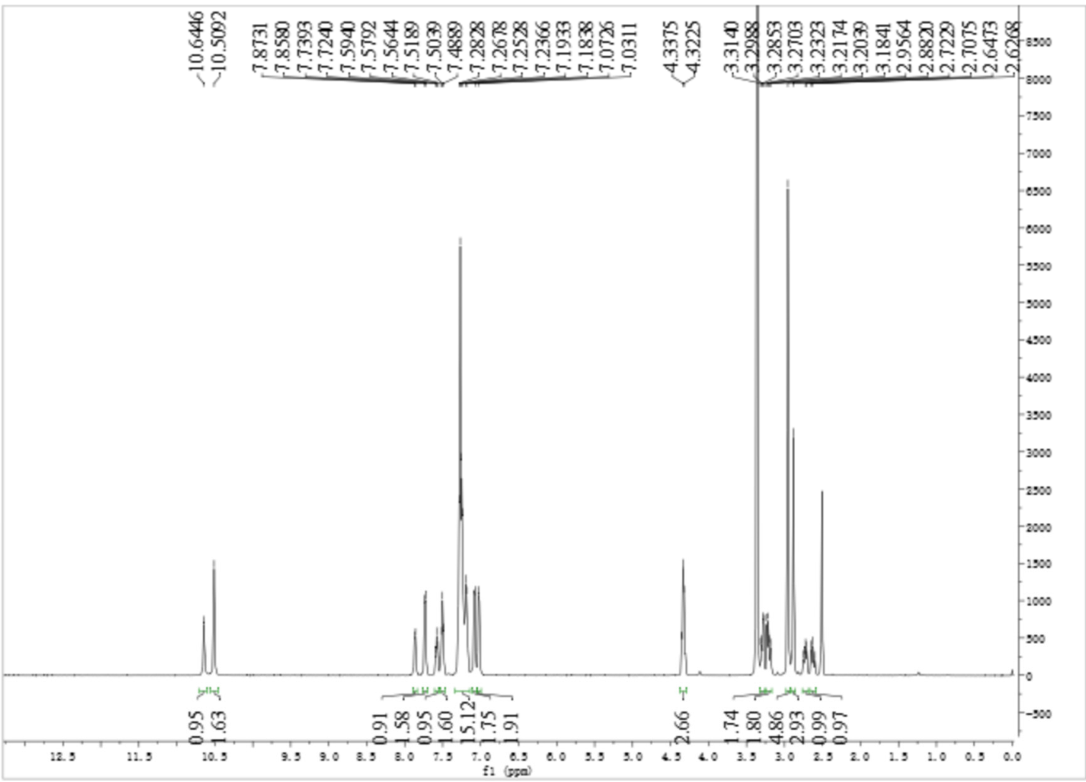

Figure S18. ^{1}H NMR (DMSO-d_6, 500 MHz) spectrum of **5a/5b**

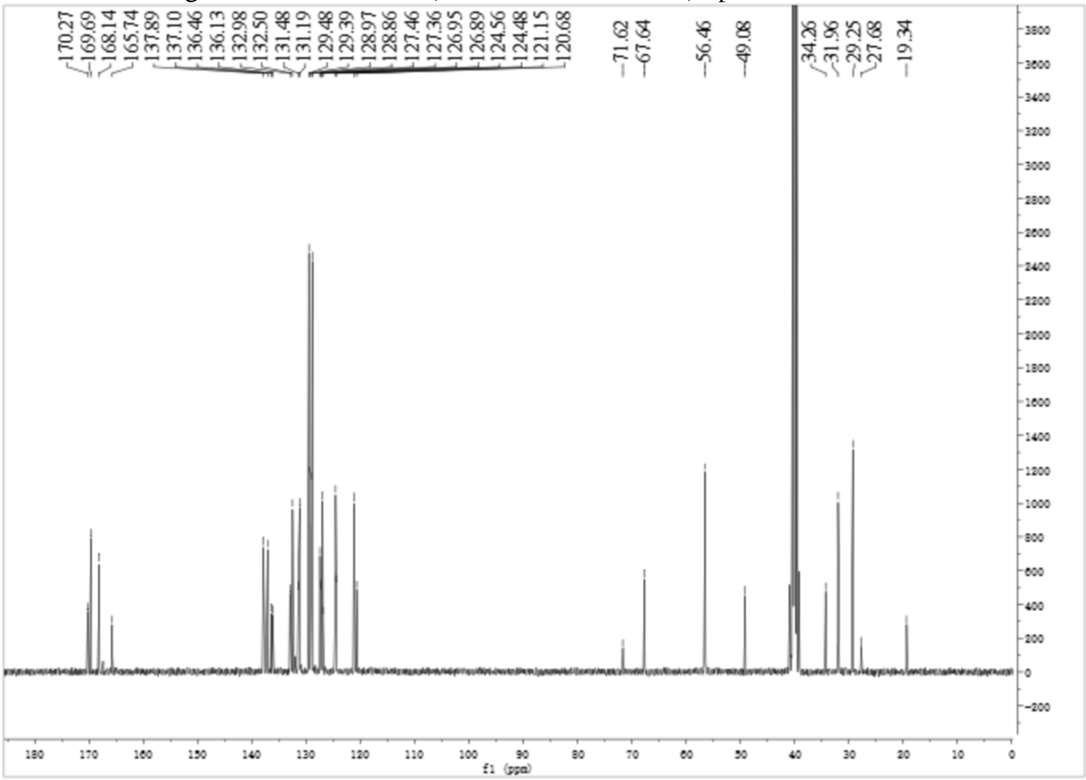

Figure S19. ^{13}C NMR (DMSO-d_6, 125 MHz) spectrum of **5a/5b**

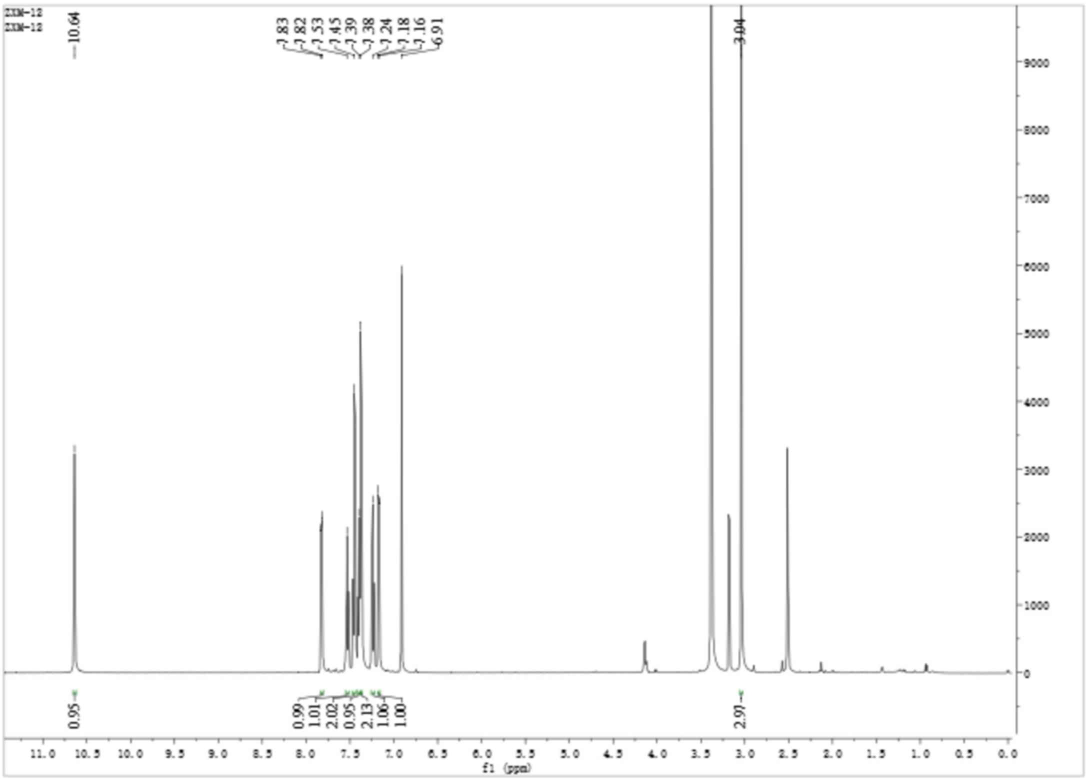

Figure S20. ^1H NMR (DMSO-d_6, 500 MHz) spectrum of **6**

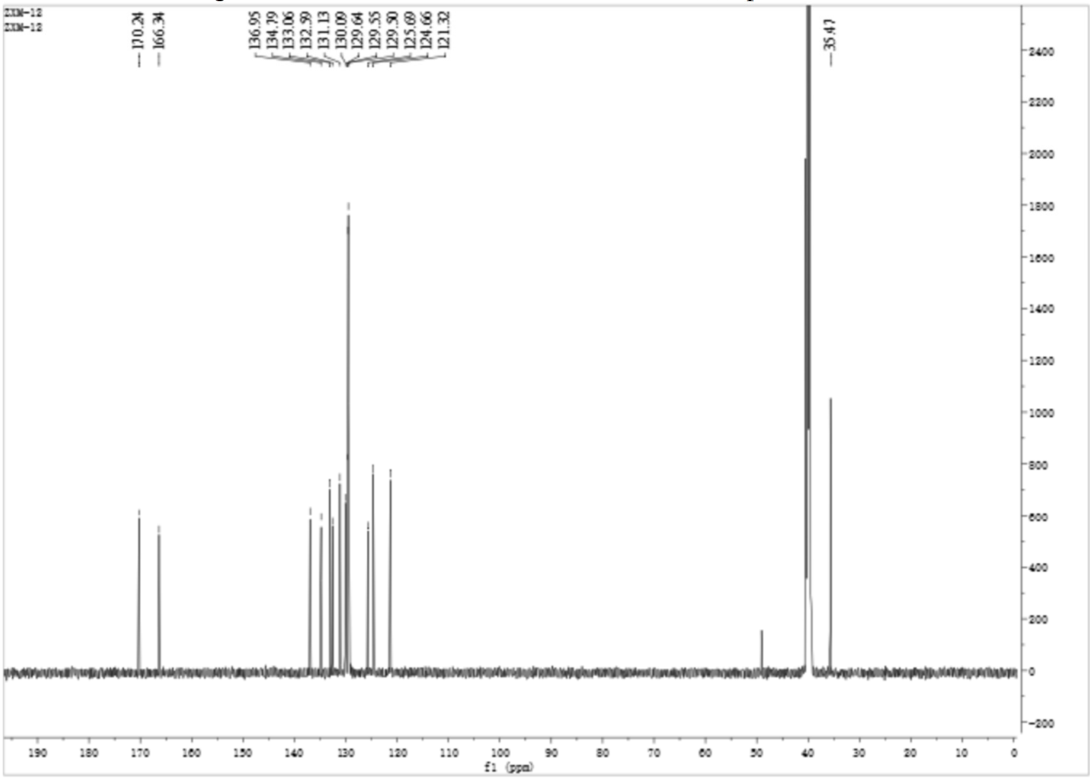

Figure S21. ^{13}C NMR (DMSO-d_6, 125 MHz) spectrum of **6**

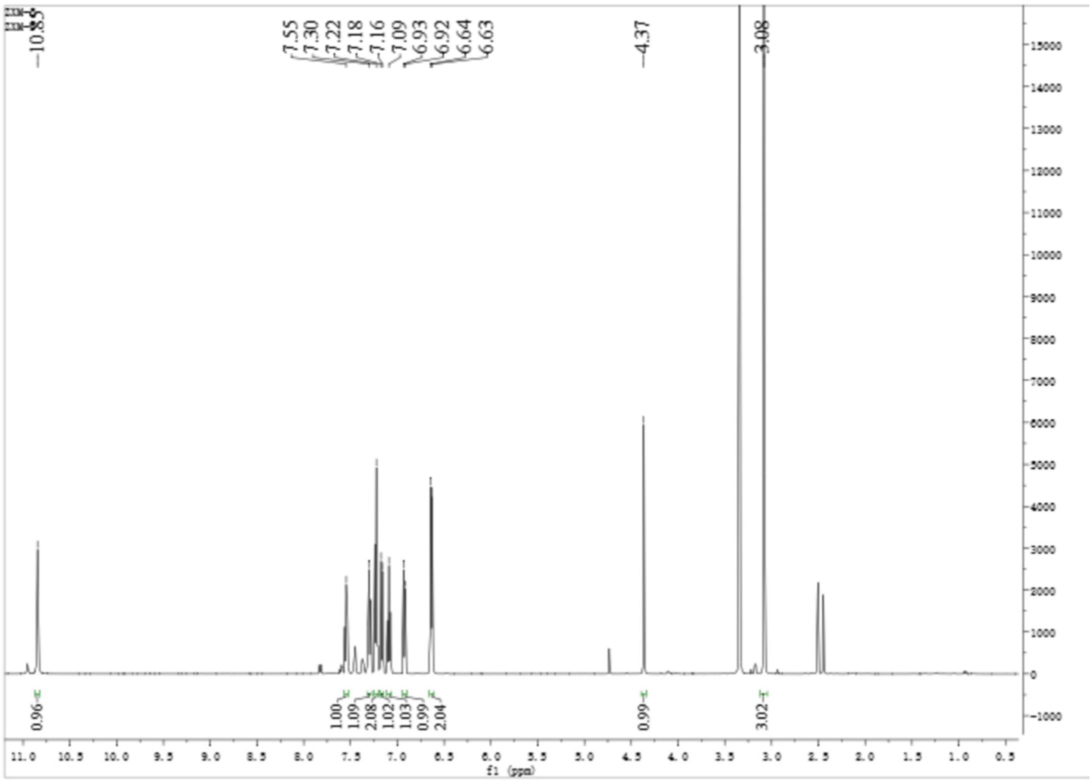

Figure S22. ^{1}H NMR (DMSO-d_6, 500 MHz) spectrum of **7**

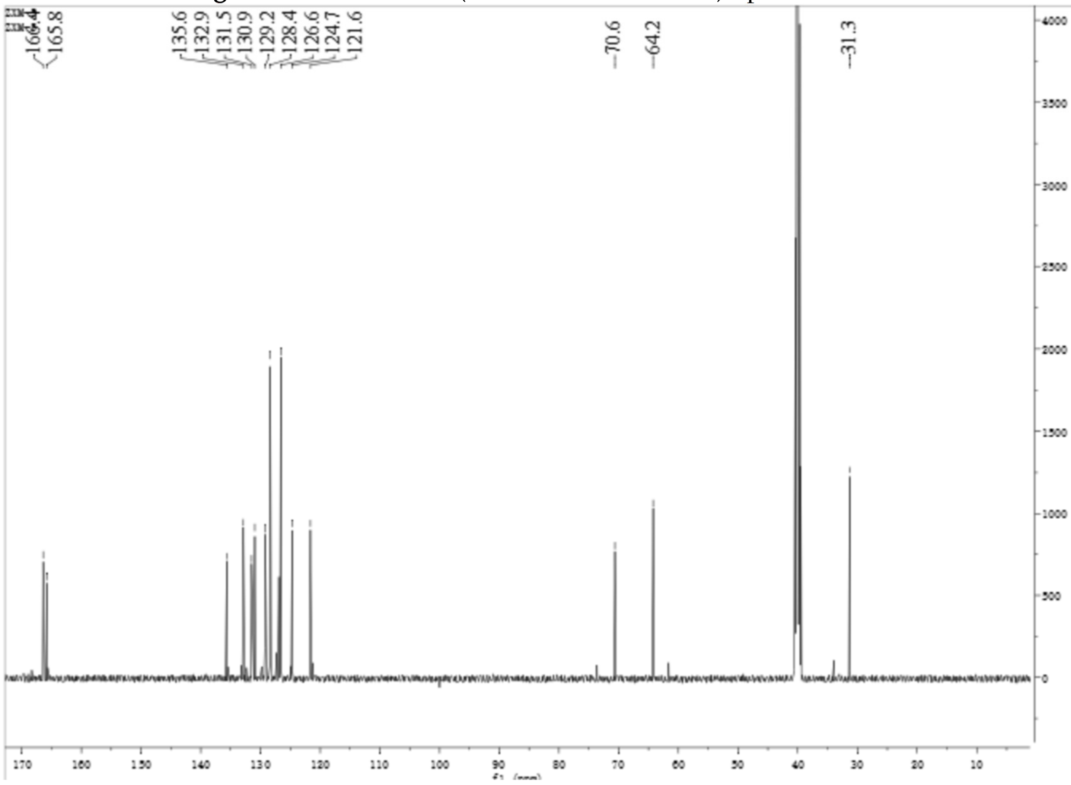

Figure S23. ^{13}C NMR (DMSO-d_6, 125 MHz) spectrum of **7**

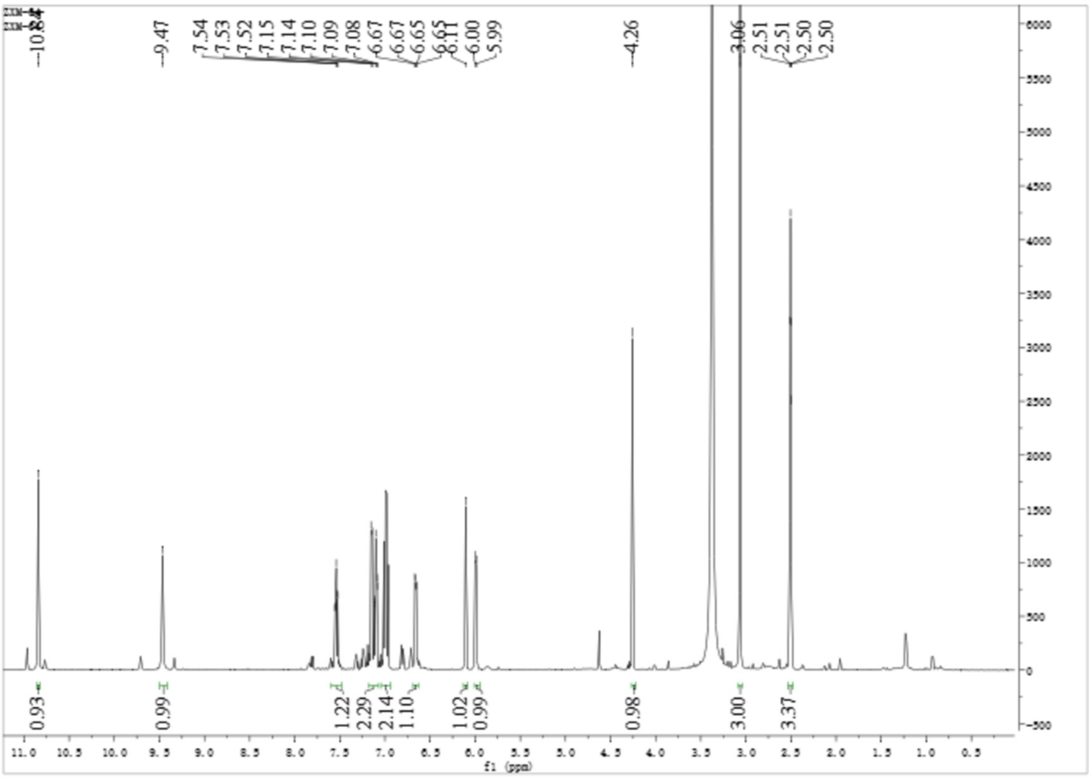

Figure S24. ^{1}H NMR (DMSO-d_6, 500 MHz) spectrum of **8**

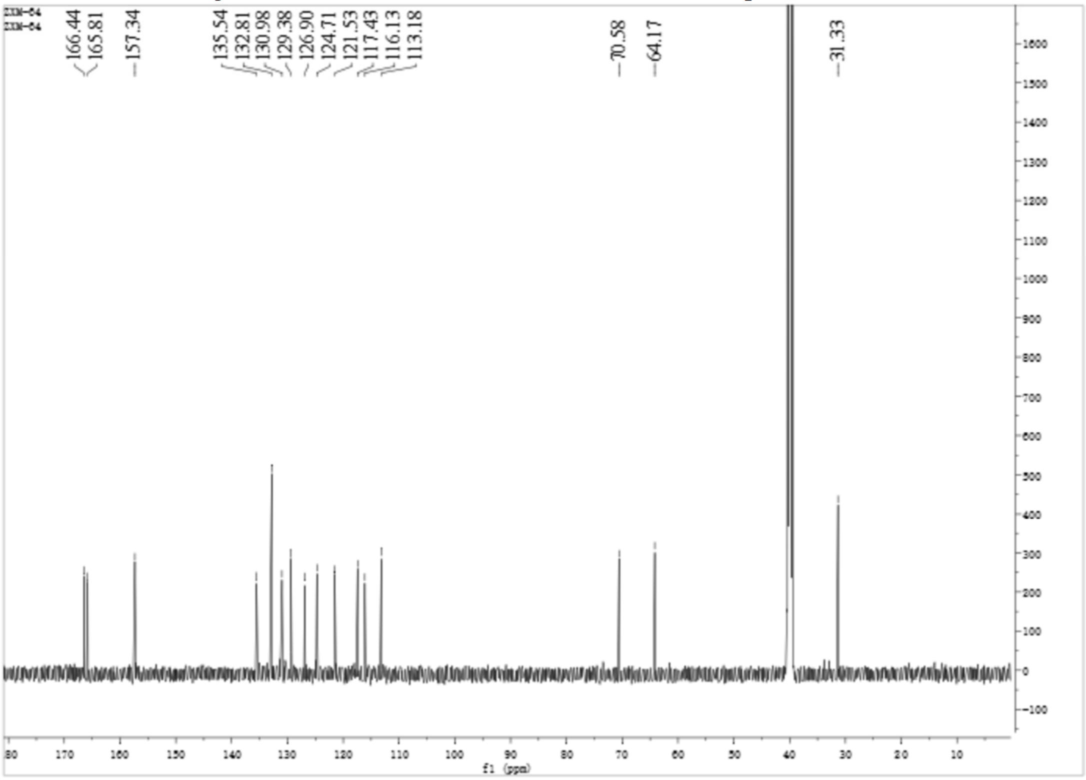

Figure S25. ^{13}C NMR (DMSO-d_6, 125 MHz) spectrum of **8**

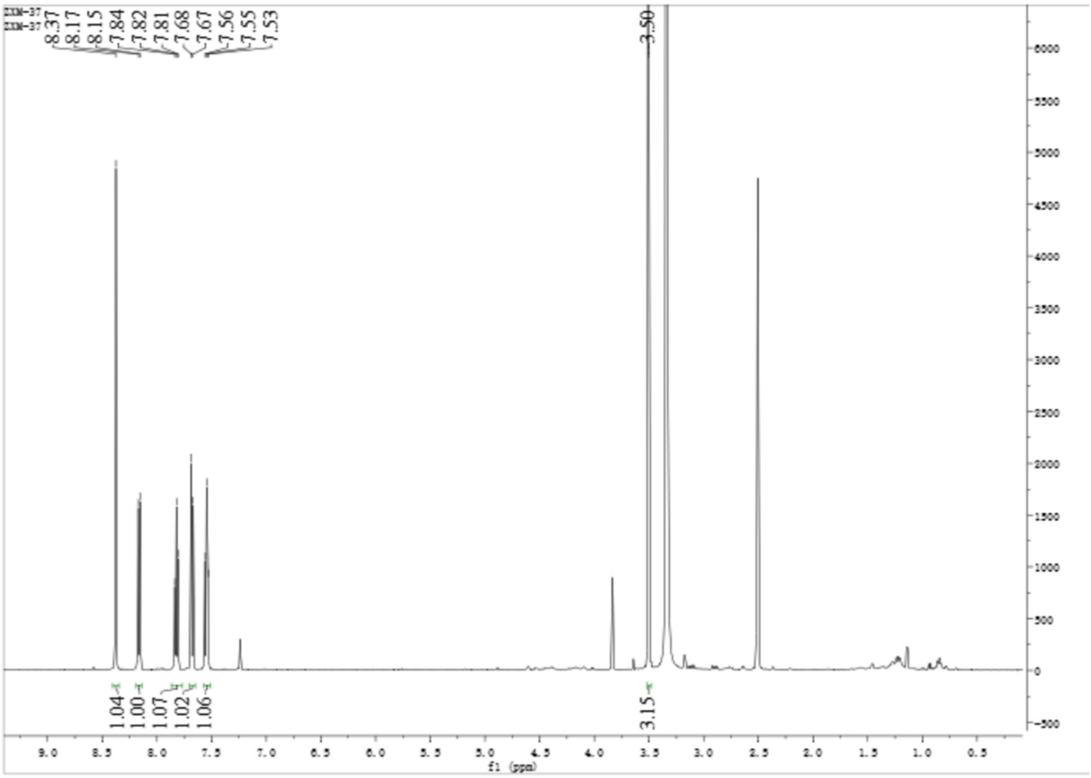

Figure S26. ^{1}H NMR (DMSO-d_6, 500 MHz) spectrum of **9**

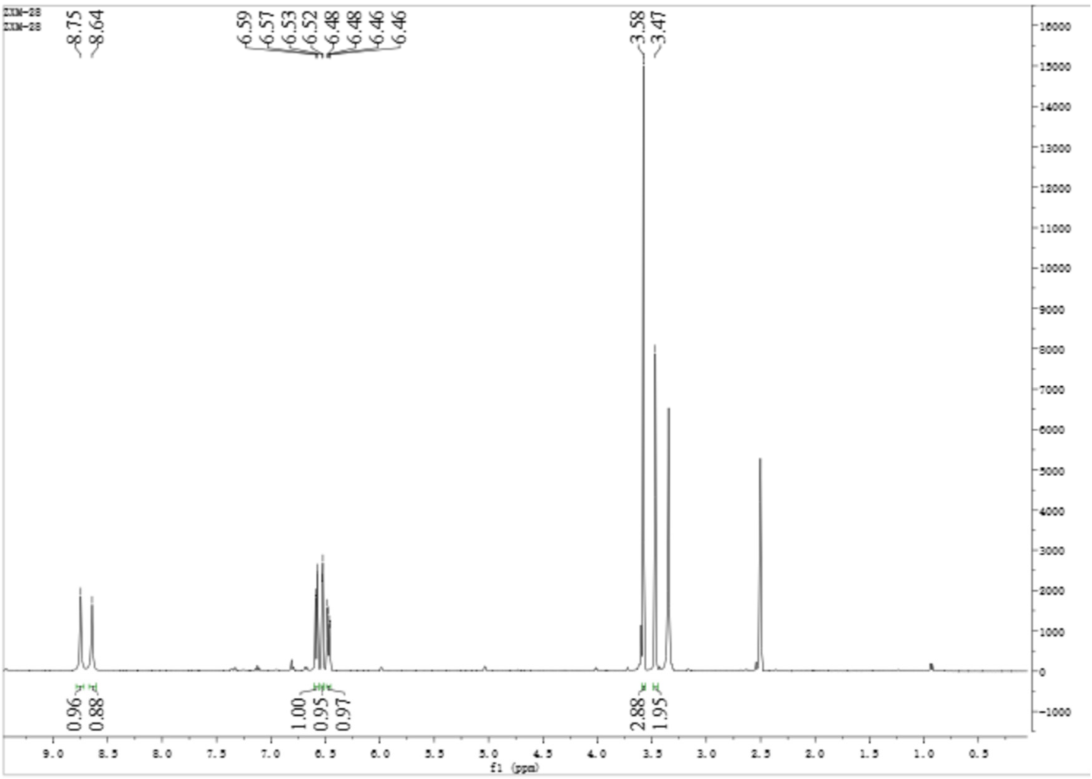

Figure S27. ^{1}H NMR (DMSO-d_6, 500 MHz) spectrum of **10**

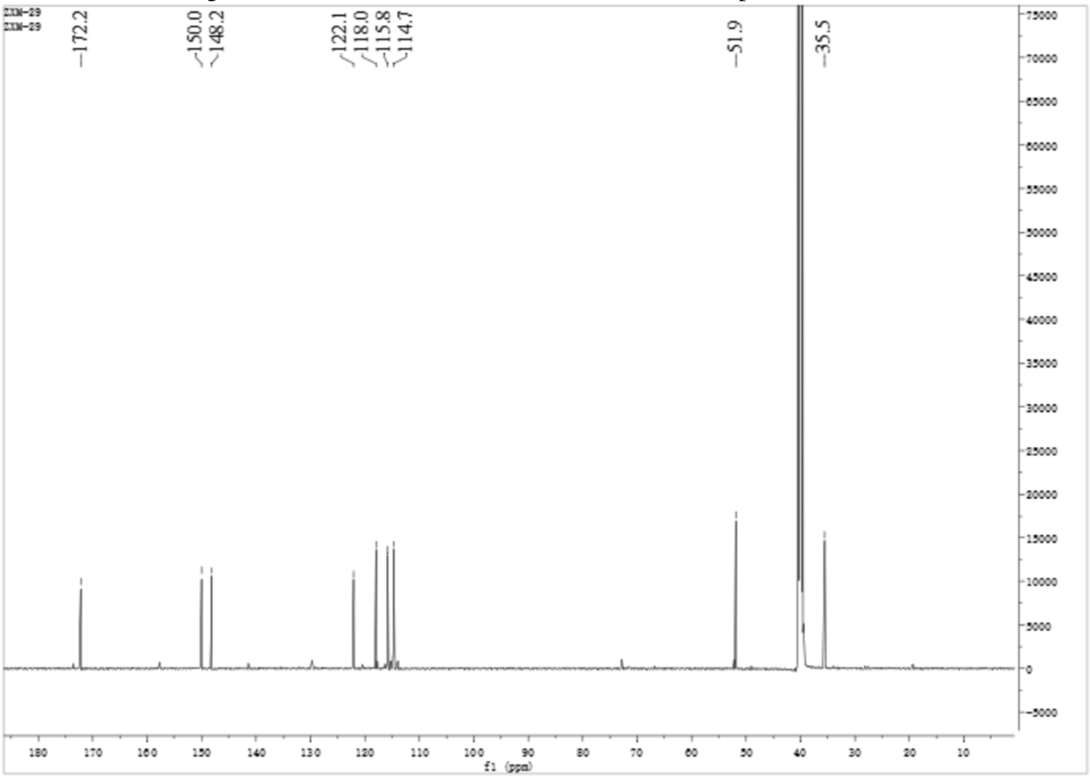

Figure S28. ^{13}C NMR (DMSO-d_6, 125 MHz) spectrum of **10**

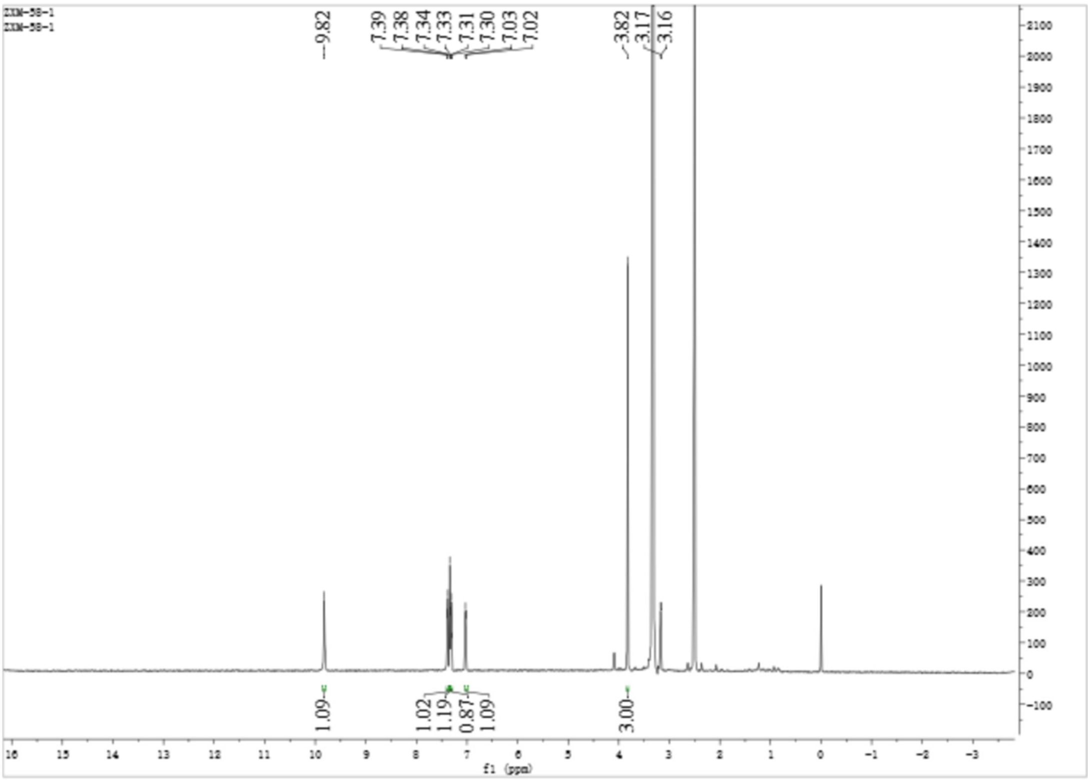

Figure S29. ^{1}H NMR (DMSO-d_6, 500 MHz) spectrum of **11**

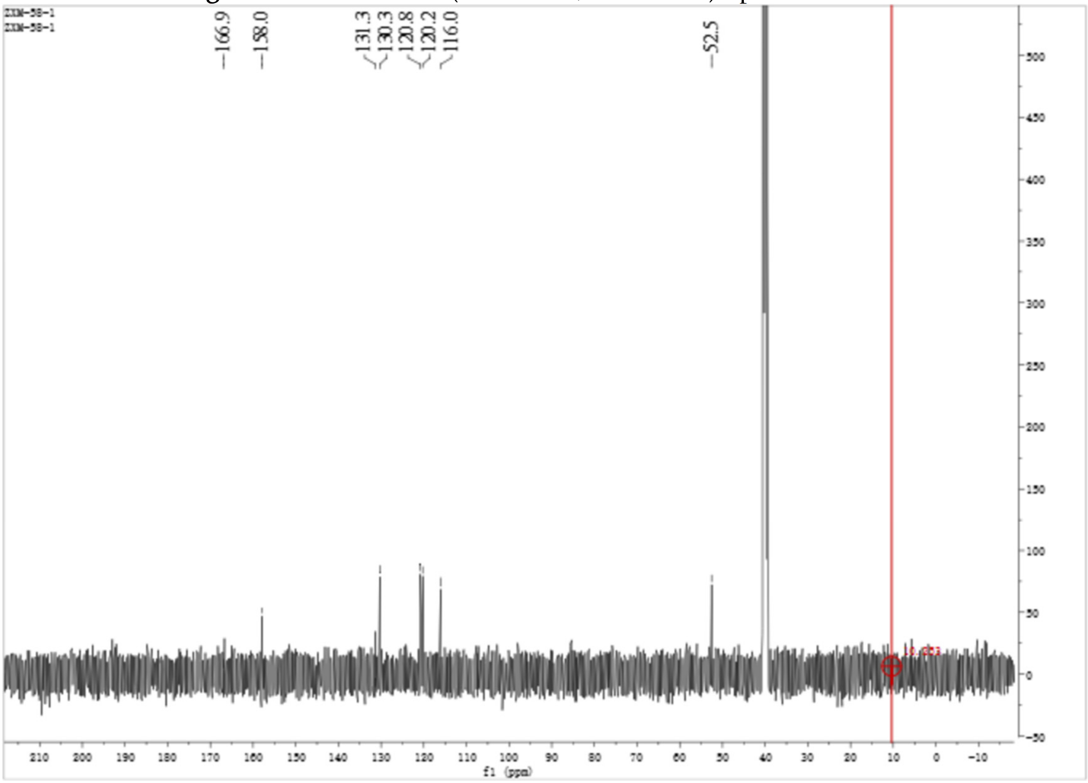

Figure S30. ^{13}C NMR (DMSO-d_6, 125 MHz) spectrum of **11**

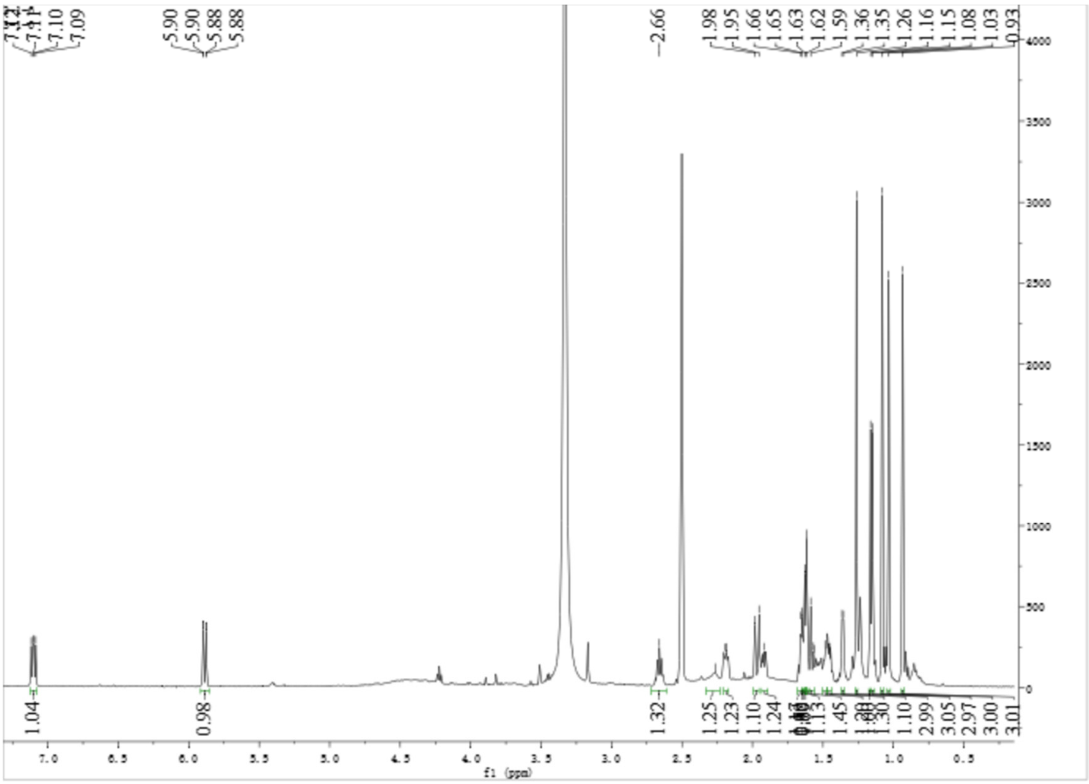

Figure S31. ^{1}H NMR (DMSO-d_6, 500 MHz) spectrum of **12**

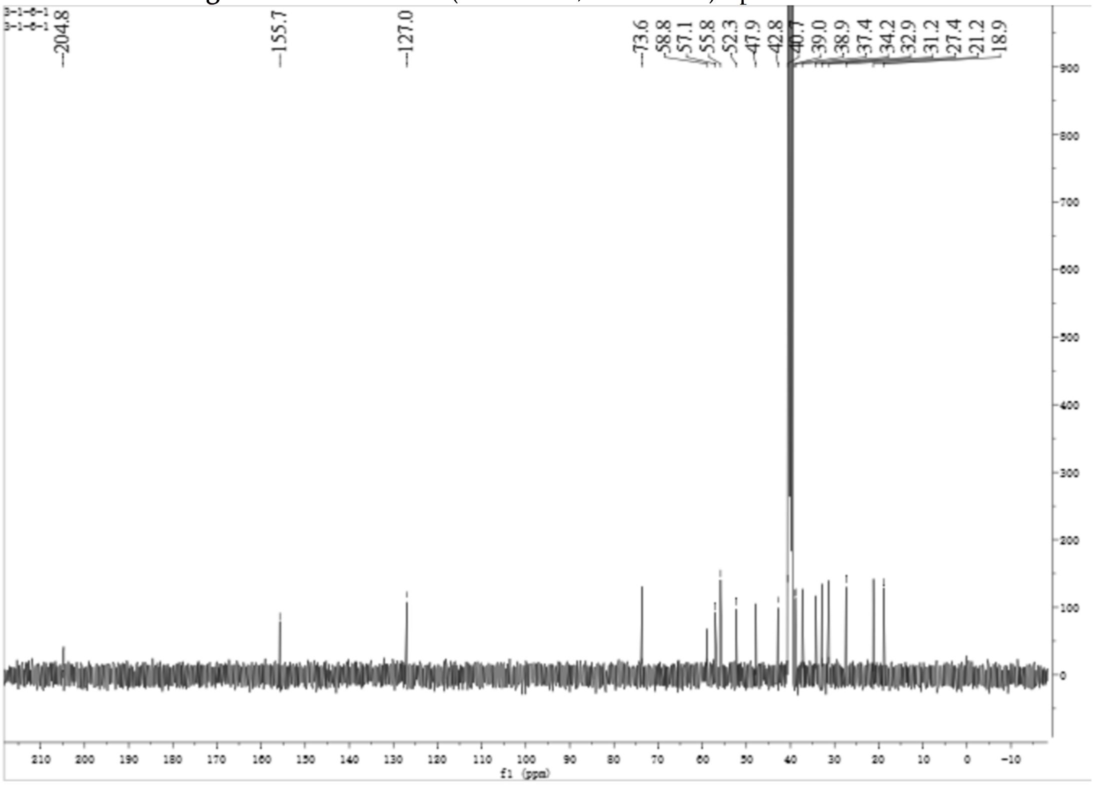

Figure S32. ^{13}C NMR (DMSO-d_6, 125 MHz) spectrum of **12**
